# Supplementary material for: VEGF-B is a novel mediator of ER stress which induces cardiac angiogenesis via RGD-binding integrins independent of VEGFR1/NRP activities
Source: Mol Ther. 2025 Mar 12;33(7):3242–56. doi: 10.1016/j.ymthe.2025.03.012 (PMC12265967; doi:10.1016/j.ymthe.2025.03.012)
Supplement: Document S1. Figures S1–S9, Tables S1 and S2, and supplemental materials and methods [file mmc1.pdf]

YMTHE, Volume *33*

Supplemental Information

VEGF-B is a novel mediator of ER stress which induces cardiac angiogenesis via RGD-binding integrins independent of VEGFR1/NRP activities

Rahul Mallick, Ahmed B. Montaser, Henna Komi, Greta Juusola, Annakaisa Tirronen, Erika Gurzeler, Maria Barbiera, Petra Korpisalo, Tetsuya Terasaki, Tiina Nieminen, and Seppo Ylä-Herttuala

Supplemental

Supplemental materials and methods

Supplemental Tables

Table S1: *Animal groups in the study*

Group	Model types	Day 0	Day 6	Number of animals		
1	VEGFR1 TK$^{-/-}$ C57BL/6J male mice (12 weeks old)	Gene transfer, LVEF measurement,	LVEF, LVESV, LVEDV, plasma protein analysis and immunohistochemistry	Ad-CMV	Ad-VEGF-B186	Ad-VEGF-B186R127S
				**5**	**5**	**5**
2	wildtype C57BL/6J male mice (12 weeks old)	Gene transfer	Immunohistochemistry	Ad-CMV	Ad-VEGF-B186	Ad-VEGF-B186R127S
				**6**	**7**	**6**
3	wildtype female domestic pigs (3 months old)	Gene transfer	Immunohistochemistry	Ad-LacZ	Ad-VEGF-B186	Ad-VEGF-B186R127S
				**6**	**6**	**6**
4	Ischemic female domestic pigs (3 months old) [Ischemia induced fourteen days before the gene transfer by placing the bottleneck stent to the left anterior descending artery in angiographic guidance]	Gene transfer	Immunohistochemistry	Ad-LacZ	Ad-VEGF-B186	Ad-VEGF-B186R127S
				**5**	**5**	**5**

Table S2: *Primer pairs for quantitative real-time PCR*

Target genes	Sequence types	Strands
**Human *GAPDH***	**Forward**	GCAAGAGCACAAGAGGAAGA (Sense)
	**Reverse**	CTACATGGCAACTGTGAGGAG (AntiSense)
**Human *HSPA5***	**Forward**	GGTGGATCACAAGGTCAAGAG (Sense)
	**Reverse**	CTACCACGCCCAGCTAATTT (AntiSense)
**Human *ANGPT2***	**Forward**	ATCAGGACACACCACGAATG (Sense)
	**Reverse**	CATCCTCACGTCGCTGAATAA (AntiSense)
**Human *VEGFA***	**Forward**	GCTTACTCTCACCTGCTTCTG (Sense)
	**Reverse**	CTGTCATGGGCTGCTTCTT (AntiSense)
**Human *ATF6***	**Forward**	GGAGCCACTGAAGGAAGATAAG (Sense)
	**Reverse**	GTGCTGCTGGAAGCAATAAAG (AntiSense)
**Human *ERN1***	**Forward**	CCTCCGAGCCATGAGAAATAA (Sense)
	**Reverse**	GGGAAGCGAGATGTGAAGTAG (AntiSense)

**Human *EIF2AK3***	**Forward**	GGAAACGAGAGCCGGATTTAT (Sense)
	**Reverse**	TATGGCAGCTTCCTGTTCTTC (AntiSense)
**Human *CSF1***	**Forward**	GGAGACCTCGTGCCAAATTA (Sense)
	**Reverse**	CGCATGGTGTCCTCCATTAT (AntiSense)
**Human *CSF2***	**Forward**	GAGCTAGAAACTCAGGATGGTC (Sense)
	**Reverse**	TCTTCTGCCATGCCTGTATC (AntiSense)
**Human *CSF3***	**Forward**	TGTGTCCTTCCCTGCATTT (Sense)
	**Reverse**	TTACCTATCTACCTCCCAGTCC (AntiSense)
**Human *XBP1***	**Forward**	GAGACAGAGAGCCAAGCTAATG (Sense)
	**Reverse**	CAGGTTCTTCCTTCACTGAGAC (AntiSense)
**Human *FLT1***	**Forward**	TGACACTTTGATCCCTGATGG (Sense)
	**Reverse**	CACAGGTCAGAAGCCCTATTT (AntiSense)
**Human *ITGAV***	**Forward**	CGACAGGCTCACATTCTACTT (Sense)
	**Reverse**	GGACTCGAGACTCCTCTTATCT (AntiSense)
**Human *ITGA5***	**Forward**	GGTGGACCAGGAAGCTATTT (Sense)
	**Reverse**	GAACCAGGTTGATCAGGTACTC (AntiSense)

Table S3: *Differential gene expression analysis and gene set enrichment analysis results*

Table S4: *Summary of DIA-based protein identification and quantification*

Supplemental Figures

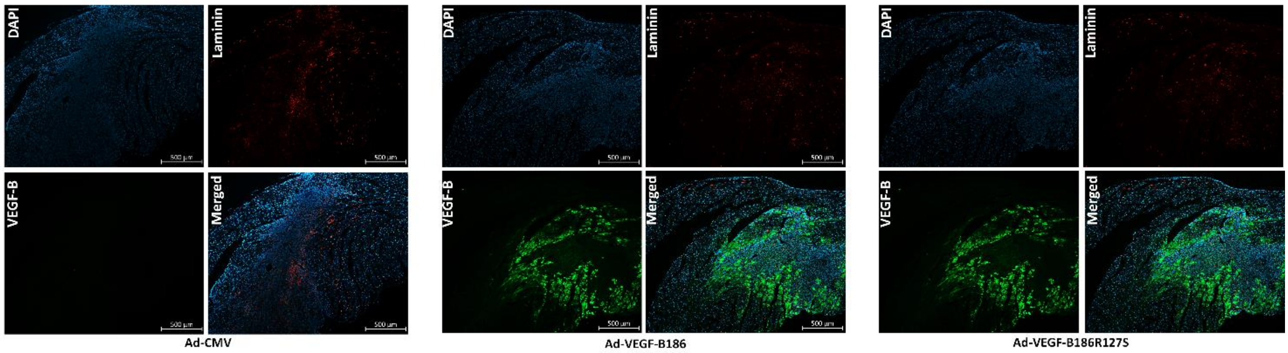

Figure S1: *VEGF-B expression in adenoviral vector transduced VEGFR1 TK $^{-/-}$ murine hearts.*

Representative images of heart tissue sections immunostained for VEGF-B, laminin, and DAPI following transduction with Ad-CMV, Ad-VEGF-B186, or Ad-VEGF-B186R127S. Scale bars: 500 µm.

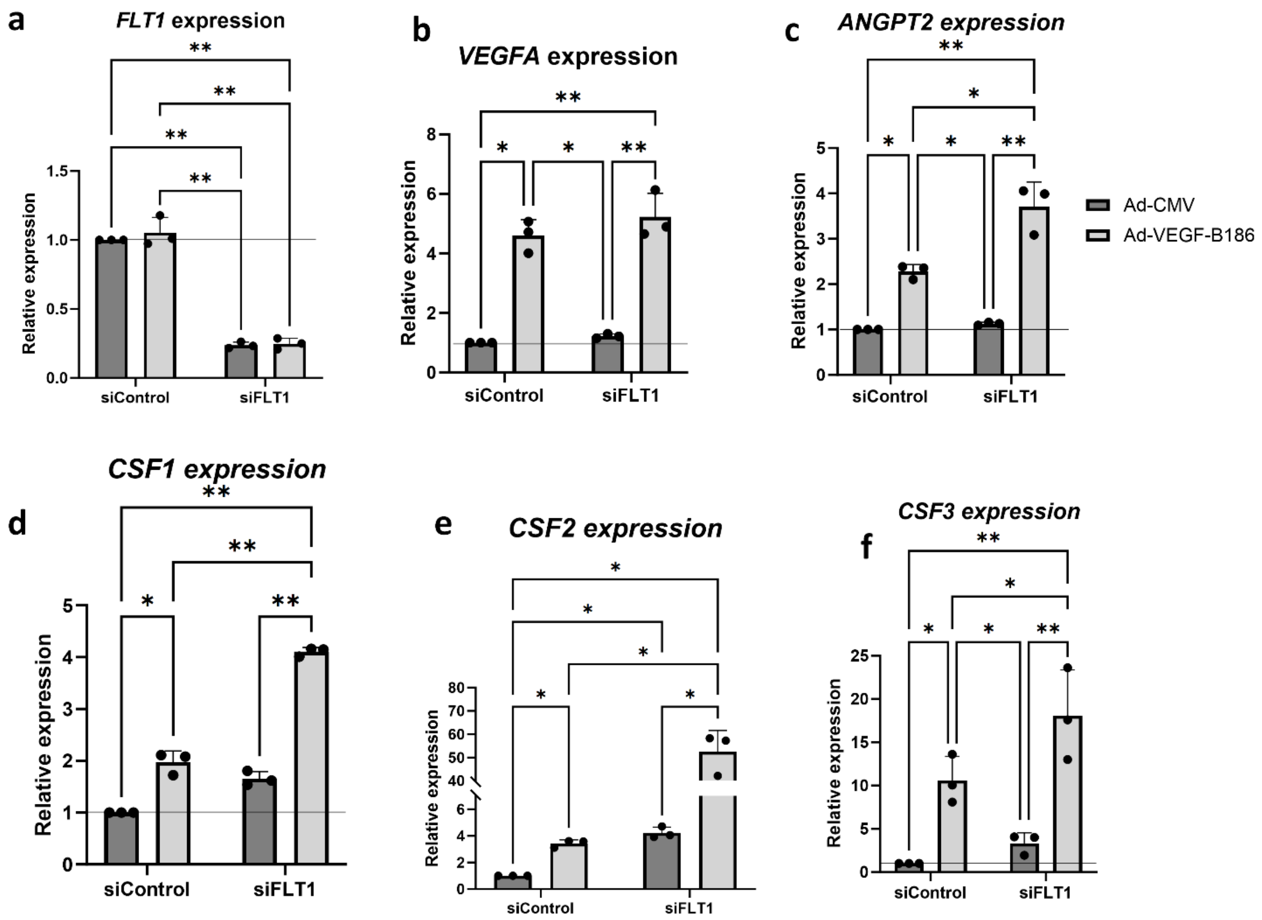

Figure S2: *VEGFR1 downstream signaling is not required for VEGF-B186-induced proangiogenic and haematopoietic growth inducing cytokine genes upregulation in cardiac microvascular endothelial cells*

a–f. Expression levels of *FLT1* (**a**), *VEGFA* (**b**), *ANGPT2* (**c**), *CSF1* (**d**), *CSF2* (**e**), and *CSF3* (**f**) in HMVEC-Cs treated with siControl (n=3) or siFLT1 (n=3) following Ad-VEGF-B186 or Ad-CMV transduction. The n value represents the number of individual cell culture experiments, which are defined as biological replicates. Horizontal bars indicate mean ± SD and *P* values versus each group by two-way ANOVA followed by Tukey's multiple comparison test. P value style: <0.05(*), <0.005(**).

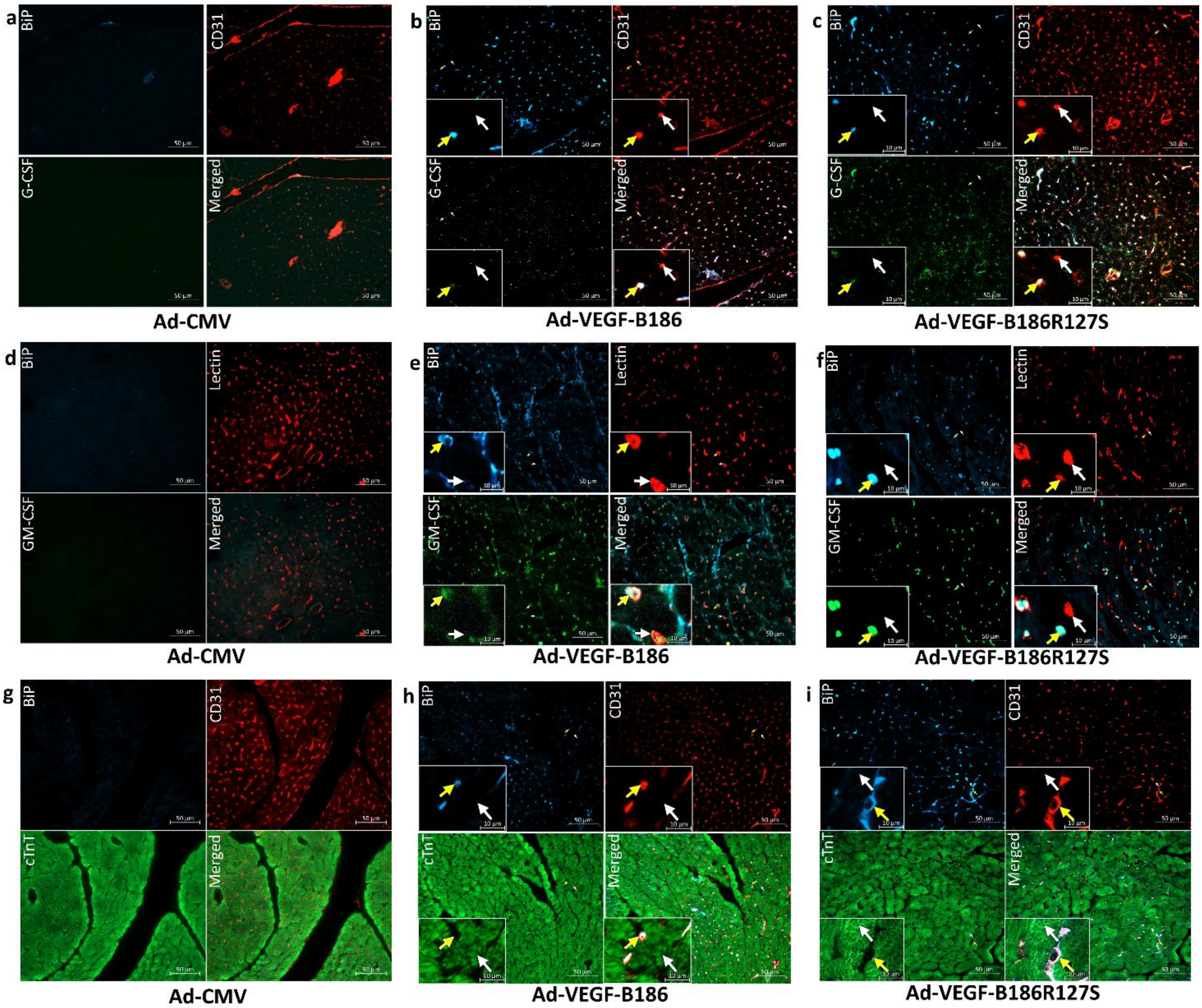

Figure S3: *Immunostained adenoviral vector transduced wildtype murine hearts.*

a–c. Representative images of sections stained for CD31, G-CSF, and BiP following Ad-CMV **(a)**, Ad-VEGF-B186 **(b)**, and Ad-VEGF-B186R127S **(c)** gene transfers. Yellow arrows indicate G-CSF–secreting, activated BiP^{+}CD31^{+} endothelial cells, while white arrows indicate quiescent CD31^{+} endothelial cells. **d–f.** Representative images of sections stained for lectin, GM-CSF, and BiP following Ad-CMV **(d)**, Ad-VEGF-B186 **(e)**, and Ad-VEGF-B186R127S **(f)** gene transfers. White arrows indicate lectin^{+} endothelial cells, while yellow arrows indicate lectin^{+}BiP^{+}GM-CSF^{+} activated endothelial cells. **g–i.** Representative images of sections stained for CD31, cTnT, and BiP following Ad-CMV **(g)**, Ad-VEGF-B186 **(h)**, and Ad-VEGF-B186R127S **(i)** gene transfers. White arrows denote cTnT^{+} cardiomyocytes, while yellow arrows denote BiP^{+}CD31^{+} endothelial cells. Scale bars: 50 µm (10 µm for the enlarged images).

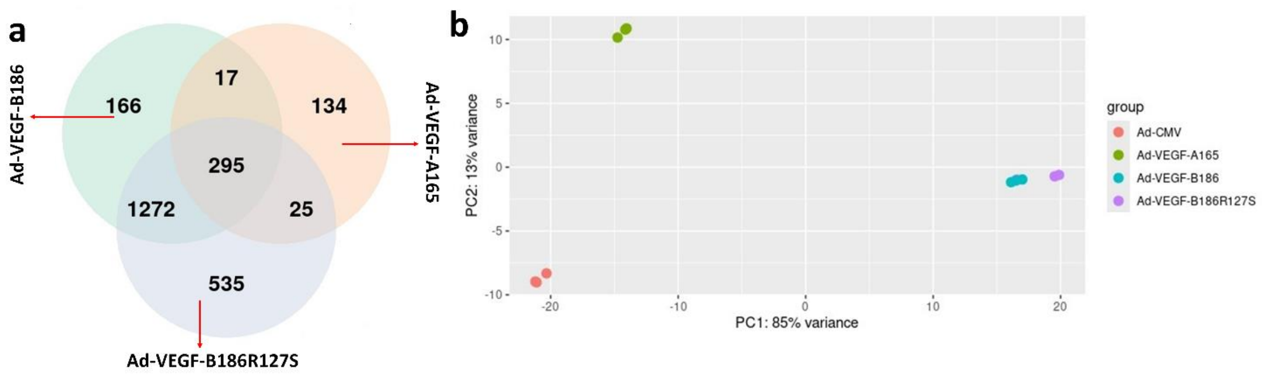

Figure S4: *Comparing differential gene expression in adenoviral vector transduced HMVEC-Cs*

Venn diagram (**a**) shows the overlap of differentially expressed genes across treatments. PCA plot (**b**) summarizes RNA-seq data distribution.

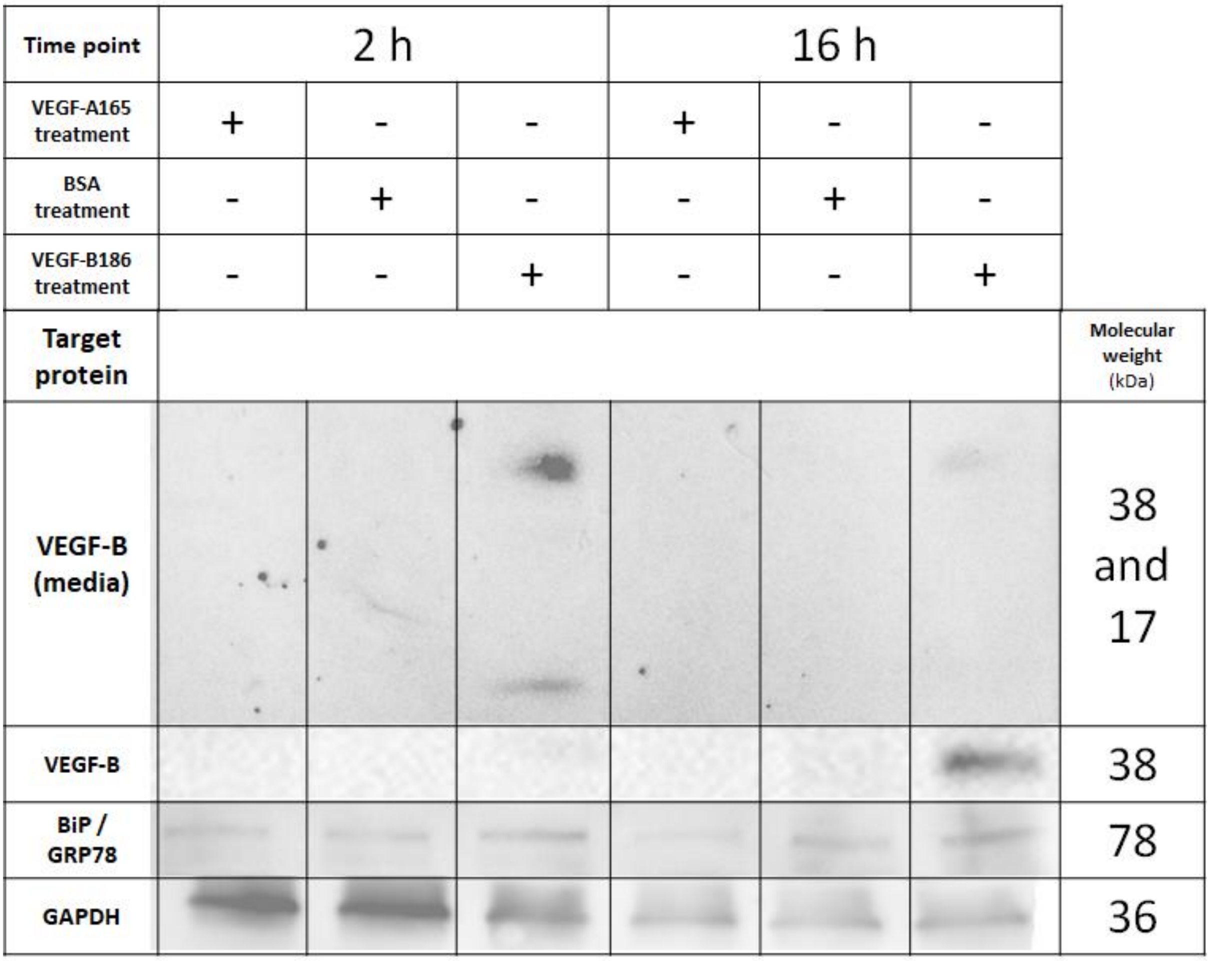

Figure S5: *rhVEGF-B186-induces expression of ER stress chaperon BiP/GRP78*

Immunoblot of VEGF-B (media and cell lysate), BiP / GRP78 and GAPDH expression in HUVECs 2 and 16 hrs post rhVEGF-B186, rhVEGF-A165 and BSA treatment

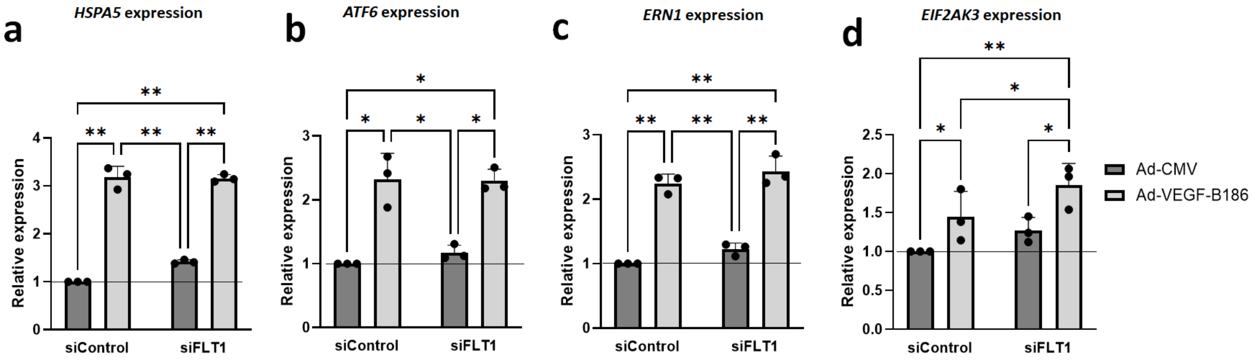

Figure S6: *VEGFR1 is dispensable for Ad-VEGF-B186-induced ER stress*

Expression of ER stress-related genes (**a-d**) is shown for siControl (n=3) or siFLT1 (n=3)-treated HMVEC-Cs post-transduction. The n value represents the number of individual cell culture experiments, which are defined as biological replicates. Horizontal bars indicate mean ± SD and *P* values versus each group by two-way ANOVA, followed by Tukey's multiple comparison test. P value style: <0.05(*), <0.005(**).

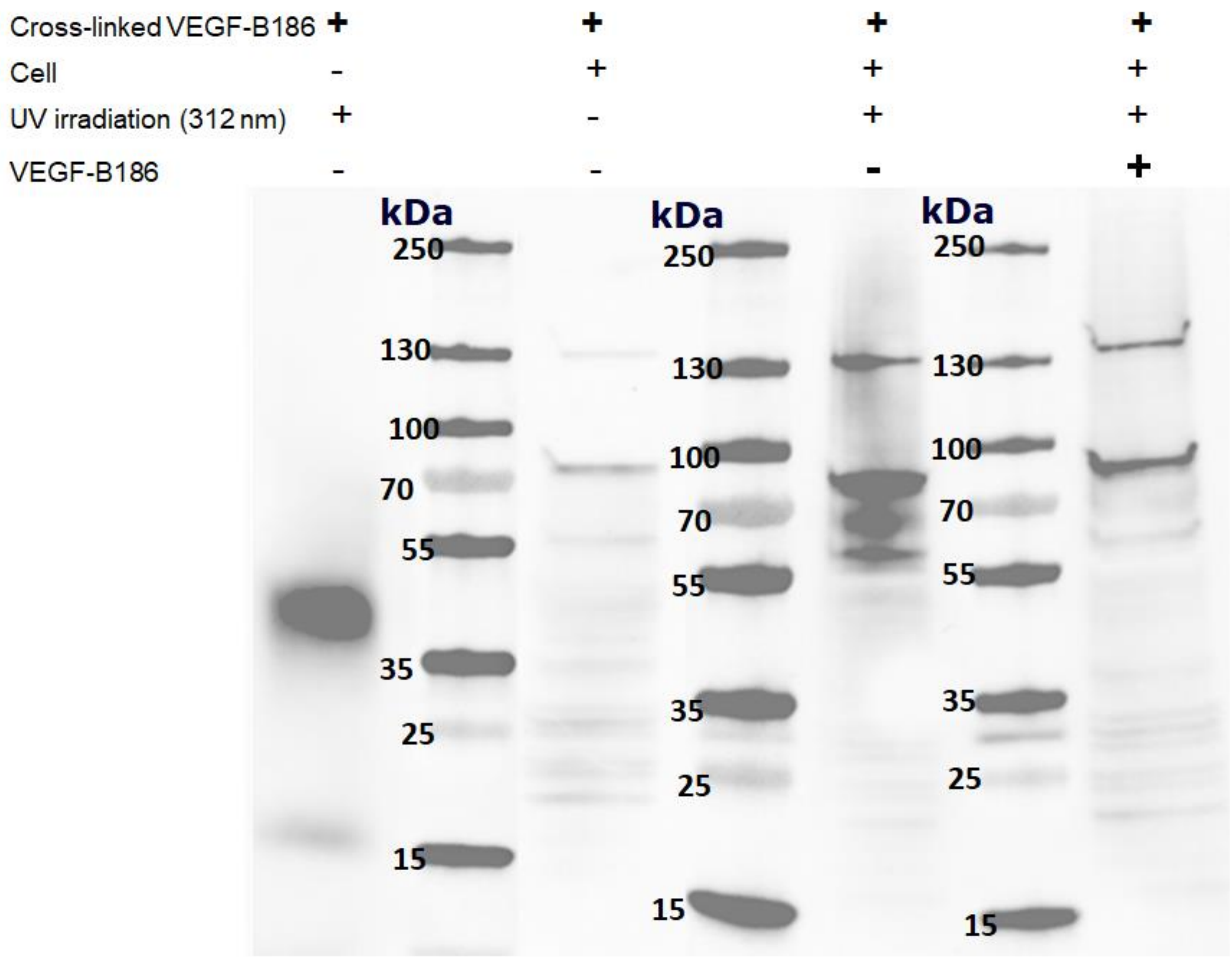

Figure S7: *The cross-linking method successfully identified the interactors of VEGF-B186.*

Biotin-labeled proteins that interact with VEGF-B186 in TeloHAECs are shown following SDS–PAGE separation and immunoblotting with HRP-conjugated streptavidin.

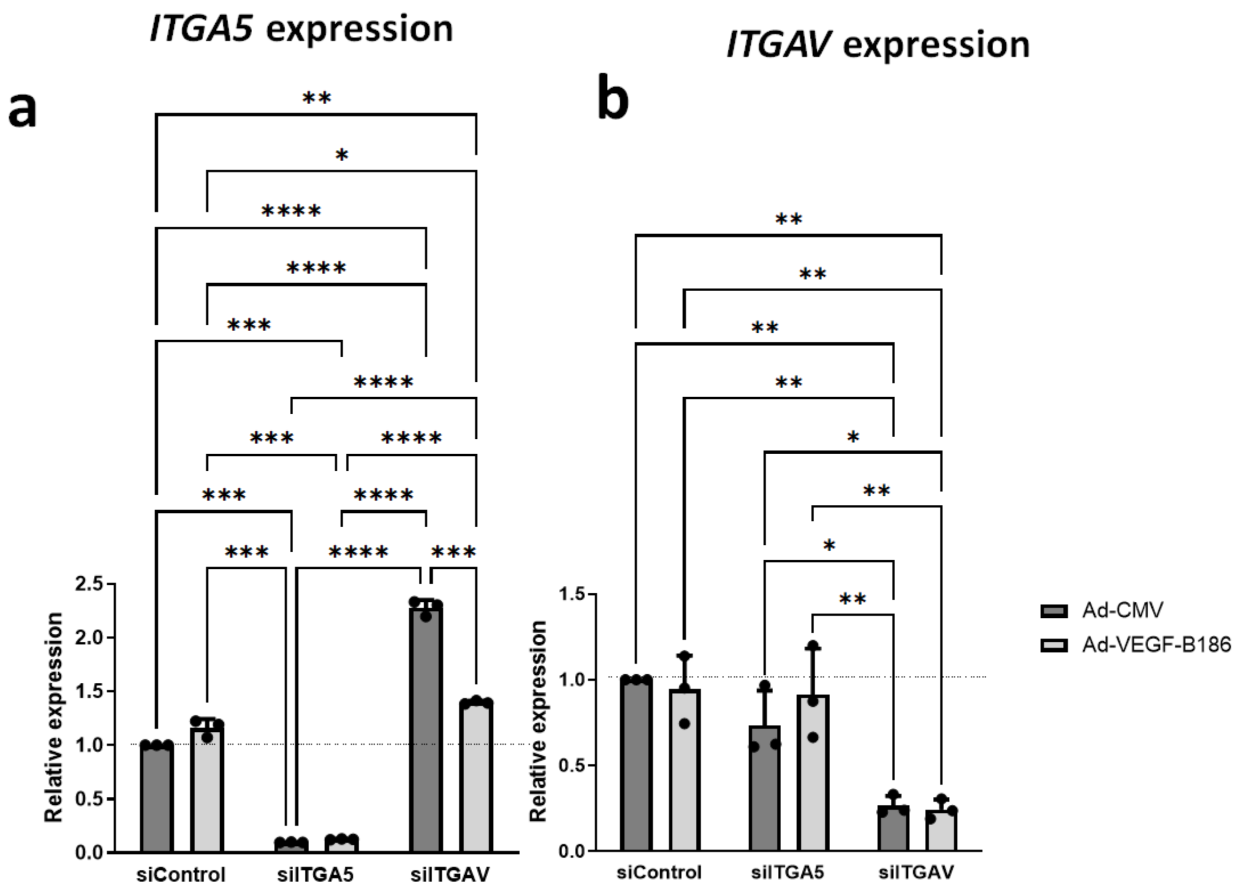

Figure S8: *ITGAV* and *ITGA5* silencing in endothelial cells.

RNA expression of *ITGA5* (**a**), and *ITGAV* (**b**) in siControl (n=3), siITGA5 (n=3) or siITGAV (n=3)-treated TeloHAECs after Ad-VEGF-B186 transduction. The n value represents the number of individual cell culture experiments, which are defined as biological replicates. Horizontal bars indicate mean ± SD and *P* values versus each group by two-way ANOVA followed by Tukey's multiple comparison test. P value style: <0.05(*), <0.005(**), <0.0005(***), <0.0001(****).

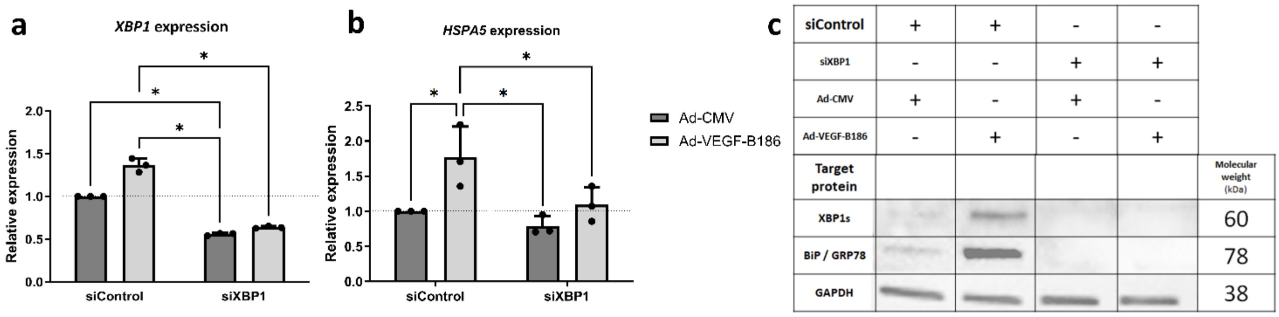

Figure S9: *The VEGF-B186-induced expression of ER stress chaperon HSPA5 is regulated by XBP1*

a,b. Graph comparing *XBP1* and *HSPA5* expression in HUVECs treated with either siControl or siXBP1 following transduction with Ad-VEGF-B186 or Ad-CMV. Horizontal bars indicate mean ± SD and *P* values versus each group by two-way ANOVA followed by Tukey's multiple comparison test. The n value represents the number of individual cell culture experiments, which are defined as biological replicates. P value style: <0.05(*), <0.005(**), <0.0005(***), <0.0001(****). **c.** Immunoblot showing the expression of XBP1s, BiP/GRP78, and GAPDH in HUVECs 24 hours after transduction with Ad-VEGF-B186, Ad-VEGF-A165, or Ad-CMV.
