# Supplementary material for: VEGF-B is a novel mediator of ER stress which induces cardiac angiogenesis via RGD-binding integrins independent of VEGFR1/NRP activities
Source: Mol Ther. 2025 Mar 12;33(7):3242–56. doi: 10.1016/j.ymthe.2025.03.012 (PMC12265967; doi:10.1016/j.ymthe.2025.03.012)
Supplement: Document S2. Article plus supplemental information [file mmc4.pdf]

Molecular Therapy
Original Article

VEGF-B is a novel mediator of ER stress which induces cardiac angiogenesis via RGD-binding integrins independent of VEGFR1/NRP activities

Rahul Mallick,[1] Ahmed B. Montaser,[2] Henna Komi,[1] Greta Juusola,[1,3] Annakaisa Tirronen,[1] Erika Gurzeler,[1] Maria Barbiera,[1] Petra Korpisalo,[3] Tetsuya Terasaki,[2] Tiina Nieminen,[1,4] and Seppo Ylä-Herttuala[1,3,4]

[1]A.I.Virtanen Institute for Molecular Sciences, Faculty of Health Sciences, University of Eastern Finland, Kuopio, Finland; [2]School of Pharmacy, Faculty of Health Sciences, University of Eastern Finland, Kuopio, Finland; [3]Heart Center and Gene Therapy Unit, Kuopio University Hospital, Kuopio, Finland

Vascular endothelial growth factor B186 (VEGF-B186), a ligand for VEGF receptor 1 (VEGFR1) and neuropilin (NRP), promotes vascular growth in healthy and ischemic myocardium. However, the mechanisms and signaling of VEGF-B186 to support angiogenesis have remained unclear. We studied the effects of VEGF-B186 and its variant, VEGF-B186R127S, which cannot bind to NRPs, using VEGFR1 tyrosine kinase knockout ($TK^{-/-}$) mice to explore the mechanism of VEGF-B186 in promoting vascular growth. Ultrasound-guided adenoviral VEGF-B186, VEGF-B186R127S, and control vector gene transfers were performed into VEGFR1 $TK^{-/-}$ mice hearts. *In vitro* studies in cardiac endothelial cells and further validation in normal and ischemic pig hearts, as well as in wild-type mice, were conducted. Both VEGF-B186 forms promoted vascular growth in VEGFR1 $TK^{-/-}$ mouse heart and increased the expression of proangiogenic and hematopoietic factors. Unlike VEGF-A, VEGF-B186 forms induced endoplasmic reticulum (ER) stress via the upregulation of Binding immunoglobulin Protein (BiP) as well as ER stress sensors (ATF6, PERK, IRE1α) through ITGAV and ITGA5 integrins, newly identified receptors for VEGF-B, activating the unfolded protein response (UPR) through XBP1. VEGFR1 and NRP are not essential for VEGF-B186-induced vascular growth. Instead, VEGF-B186 can stimulate cardiac regeneration through RGD-binding integrins and ER stress, suggesting a novel mechanism of action for VEGF-B186.

INTRODUCTION

Arteries, veins, and capillaries form the vascular network, and the stability of the vessels is ensured by recruited mural cells, such as pericytes and smooth muscle cells.[1] When the blood flow is interrupted, the nutrition and oxygen supply as well as CO_2 and waste removal are hindered in the ischemic tissue. One of the most affected organs in the human body due to blood perfusion defects is the heart. Reduced perfusion is usually caused by coronary heart disease, which is the major cause of mortality.[2] Presently, catheter-assisted reperfusion, stenting, bypass surgery, and thrombolysis of the occluded coronary vessels are the primary treatment options.[3,4] Due to the limited regeneration capacity of adult cardiomyocytes, timely reperfusion of the myocardial tissue is crucial.[3] Despite the current reperfusion strategies, insufficient blood flow or no-flow situation occurs frequently.[5] Thus, there is a clear need to develop efficient and minimally invasive procedures for the treatment of these patients.

As members of the vascular endothelial growth factor (VEGF) family are well-known regulators of blood vessel growth, several attempts have been made to induce angiogenesis in ischemic myocardium.[6–12] Earlier studies have shown that VEGF-B186 gene transfer into the myocardium in rodent and pig models results in the growth of coronary vasculature along with improved perfusion.[13–18] We have shown that only the full-length form of VEGF-B186 induces angiogenesis but the proteolytically cleaved N-terminal domain of the VEGF-B186 does not, despite binding to VEGFR1 (encoded by *FLT1*).[17] This study aims to further explore the mechanism of VEGF-B186-induced angiogenesis as well as the roles of neuropilin (NRP) and vascular endothelial growth factor receptor 1 (VEGFR1) in mediating VEGF-B186 effects in the heart. To ensure that the effects of VEGF-B186 were not influenced by its binding to NRP after proteolytic cleavage, we used a mutant version of the full-length VEGF-B186, known as VEGF-B186R127S, which does not bind to NRP.[17] This allowed us to specifically assess the impact of the full-length form of VEGF-B186 on the study outcomes. Since adeno-associated virus-based VEGF-B gene transfer has shown to induce cardiac hypertrophy after long-term expression, we opted for adenovirus (Ad)-based VEGF-B gene therapy due to its strong but transient expression.[19,20]

Received 9 October 2024; accepted 6 March 2025;
https://doi.org/10.1016/j.ymthe.2025.03.012.

[4]These authors contributed equally

Correspondence: Seppo Ylä-Herttuala, MD, PhD, FESC, A.I.Virtanen Institute for Molecular Sciences, University of Eastern Finland, 70210 Kuopio, Finland.
E-mail: seppo.ylaherttuala@uef.fi

 © 2025 The Author(s).
Published by Elsevier Inc. on behalf of The American Society of Gene and Cell Therapy.
This is an open access article under the CC BY license (http://creativecommons.org/licenses/by/4.0/).

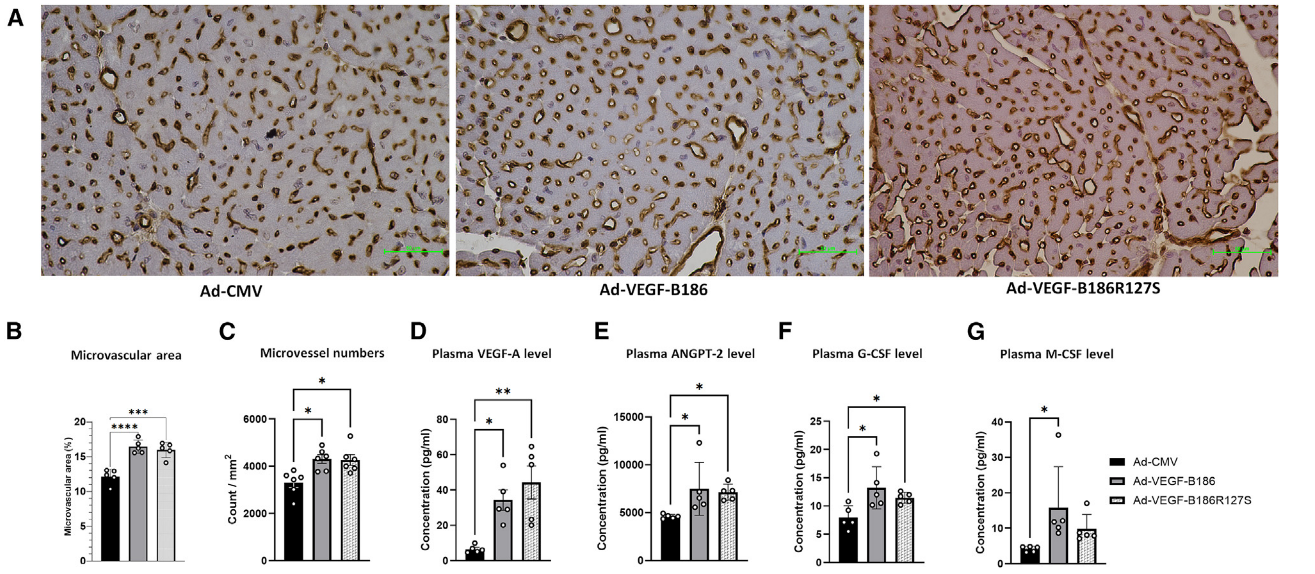

Figure 1. Myocardial vascular growth via the upregulation of proangiogenic factors and hematopoietic growth-inducing cytokines in VEGFR1 TK$^{-/-}$ mice

Representative images of CD31-stained heart tissue sections from VEGFR1 TK$^{-/-}$ mice 6 days after gene transfer (A). Scale bars, 50 μm. Quantification of microvascular area (B) and microvessel numbers (C) in heart tissues. Plasma levels of VEGF-A (D), ANGPT-2 (E), G-CSF (F), and M-CSF (G) are shown for mice treated with Ad-CMV, Ad-VEGF-B186, and Ad-VEGF-B186R127S. Each dot indicates one mouse, which is defined as a biological replicate; $N = 5$ in Ad-VEGF-B186, $N = 5$ in Ad-VEGF-B186R127S, and $N = 5$ in Ad-CMV groups. Horizontal bars indicate mean ± SD and p values vs. each group by one-way ANOVA, followed by Dunnett's multiple comparison test. ***$p < 0.0005$, ****$p < 0.0001$.

RESULTS

Neither VEGFR1 nor NRP are necessary for Ad-VEGF-B-induced microvascular growth in the heart

Microvascular growth was analyzed in VEGFR1 tyrosine kinase knockout (TK$^{-/-}$) mouse myocardium 6 days after the gene transfers. Overexpression of VEGF-B186R127S and VEGF-B186 led to a significantly increased total microvascular area in the mouse heart (Figures 1 and S1). CD31-stained total microvascular area and microvessel numbers in the Ad-VEGF-B186R127S and Ad-VEGF-B186 injected regions were increased 1.3-fold compared with the control (Ad-CMV) (Figures 1B and 1C). As proteolytically resistant VEGF-B186R127S only binds to VEGFR1 and VEGFR1 TK$^{-/-}$ mice are ablated of the VEGFR1 downstream signaling,[18,21] neither VEGFR1 nor NRP are required for the VEGF-B-induced microvascular growth.

Ad-VEGF-B gene therapy induces the proliferation of cells other than cardiomyocytes in the heart without VEGFR1 or NRP contribution

We found that VEGF-B186 gene therapy increased the number of proliferating cells in the heart without downstream signaling of VEGFR1 (Figures 2A–2C). Even NRP binding was dispensable for inducing cell proliferation in the heart of the VEGFR1 TK$^{-/-}$ mouse as VEGF-B186R127S showed similar effects as VEGF-B186 (Figures 2A–2C). Interestingly, the proliferating cells were not cardiomyocytes, and they were located at or around blood vessels (Figures 2D–2I). Plasma membrane stain laminin verified that Ki67$^+$ proliferating cells were small and not co-expressing cardiac TnT (cTnT) but were either CD31$^+$ endothelial cells or CD31$^-$ other cell types (Figures 2D–2I). We showed here that a significant number of these proliferating cells (Figure 2A) were PDGFR-β$^+$c-kit$^+$ mesenchymal stromal cells (Figures 2C and 2J–2L).

Ablation of VEGFR1 signaling does not inhibit Ad-VEGF-B-induced endothelial activation

Intramyocardial Ad-VEGFB186R127S and Ad-VEGFB186 injections were shown to significantly upregulate VEGF-A and angiopoietin-2 (ANGPT2) levels in VEGFR1 TK$^{-/-}$ mouse plasma 6 days after the gene transfers (Figures 1D and 1E). ANGPT2 has been identified as a potent proangiogenic factor that functions in collaboration with VEGF-A during endothelial activation.[22,23] Thus, VEGF-B186R127S, like the native VEGF-B186 isoform, activates endothelial cells in the heart to contribute to the microvascular growth without the VEGFR1 signaling. Consistent with the *in vivo* data, we verified significant upregulation of *VEGF-A* and *ANGPT2* in Ad-VEGF-B186 transduced *FLT1*-depleted human cardiac microvascular endothelial cells (HMVEC-Cs) (Figures S2A–S2C).

Ablation of VEGFR1 signaling does not affect VEGF-B-mediated hematopoietic growth-inducing cytokine secretion

To understand the cause of endothelial and mesenchymal stromal cell proliferation, we investigated cytokine production in the heart. We found that intramyocardial Ad-VEGF-B186R127S significantly upregulated G-CSF levels, while Ad-VEGF-B186 injections upregulated both G-CSF and M-CSF levels in VEGFR1 TK$^{-/-}$ mouse plasma 6 days following the gene transfers (Figures 1F and 1G). A similar

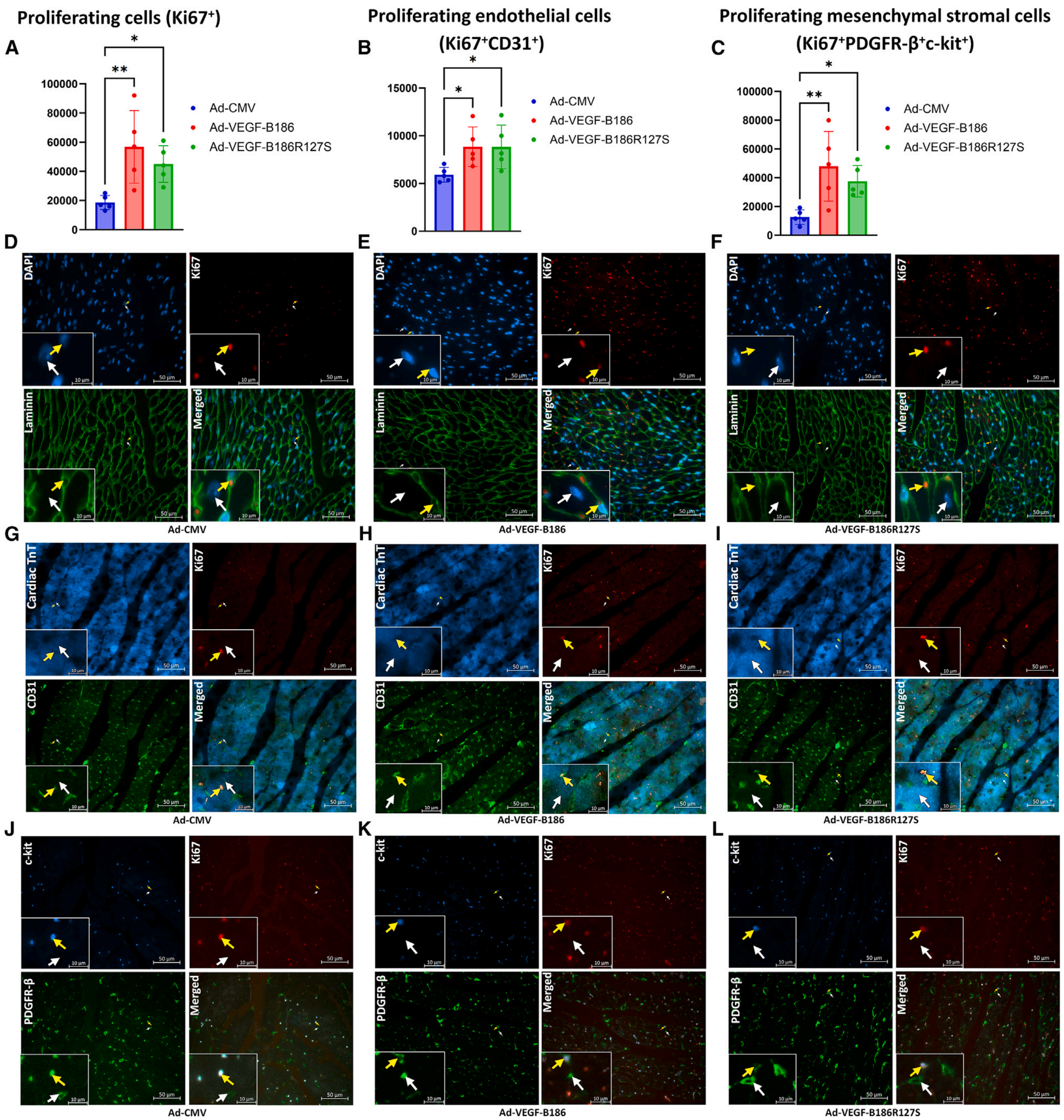

Figure 2. Proliferating cells in the adenoviral vector injected hearts of VEGFR1 TK $^{-/-}$ mice

(A) Quantification of Ki67^{+} proliferating cells. (B) Quantification of Ki67^{+}CD31^{+} proliferating endothelial cells. (C) Quantification of Ki67^{+}PDGFR-β^{+}c-kit^{+} proliferating mesenchymal stromal cells. Data represent five mice per group; horizontal bars indicate mean ± SD. Statistical significance was determined by one-way ANOVA with Dunnett's multiple comparison test (*$p < 0.05$, **$p < 0.005$). (D–F) Representative images of laminin and Ki67 immunostained heart tissue sections following Ad-CMV (D), Ad-VEGF-B186 (E), and Ad-VEGF-B186R127S (F) gene transfers. White arrows highlight cardiomyocyte nuclei; yellow arrows indicate Ki67^{+} proliferating non-cardiomyocytes. (G–I) Representative images of triple immunostaining for CD31, cTnT, and Ki67 following Ad-CMV (G), Ad-VEGF-B186 (H), and Ad-VEGF-B186R127S (I) gene transfers. White arrows denote Ki67^{+} proliferating cells that are neither cardiomyocytes nor CD31^{+} cells; yellow arrows indicate Ki67^{+}CD31^{+} proliferating endothelial cells. (J–L) Representative images of PDGFR-β, c-kit, and Ki67 immunostained heart tissue sections following Ad-CMV (J), Ad-VEGF-B186 (K), and Ad-VEGF-B186R127S (L) gene transfers. Yellow arrows point to Ki67^{+}PDGFR-β^{+}c-kit^{+} mesenchymal stromal cells; white arrows denote PDGFR-β^{+} quiescent mural cells. Scale bars, 50 μm (10 μm for the enlarged images).

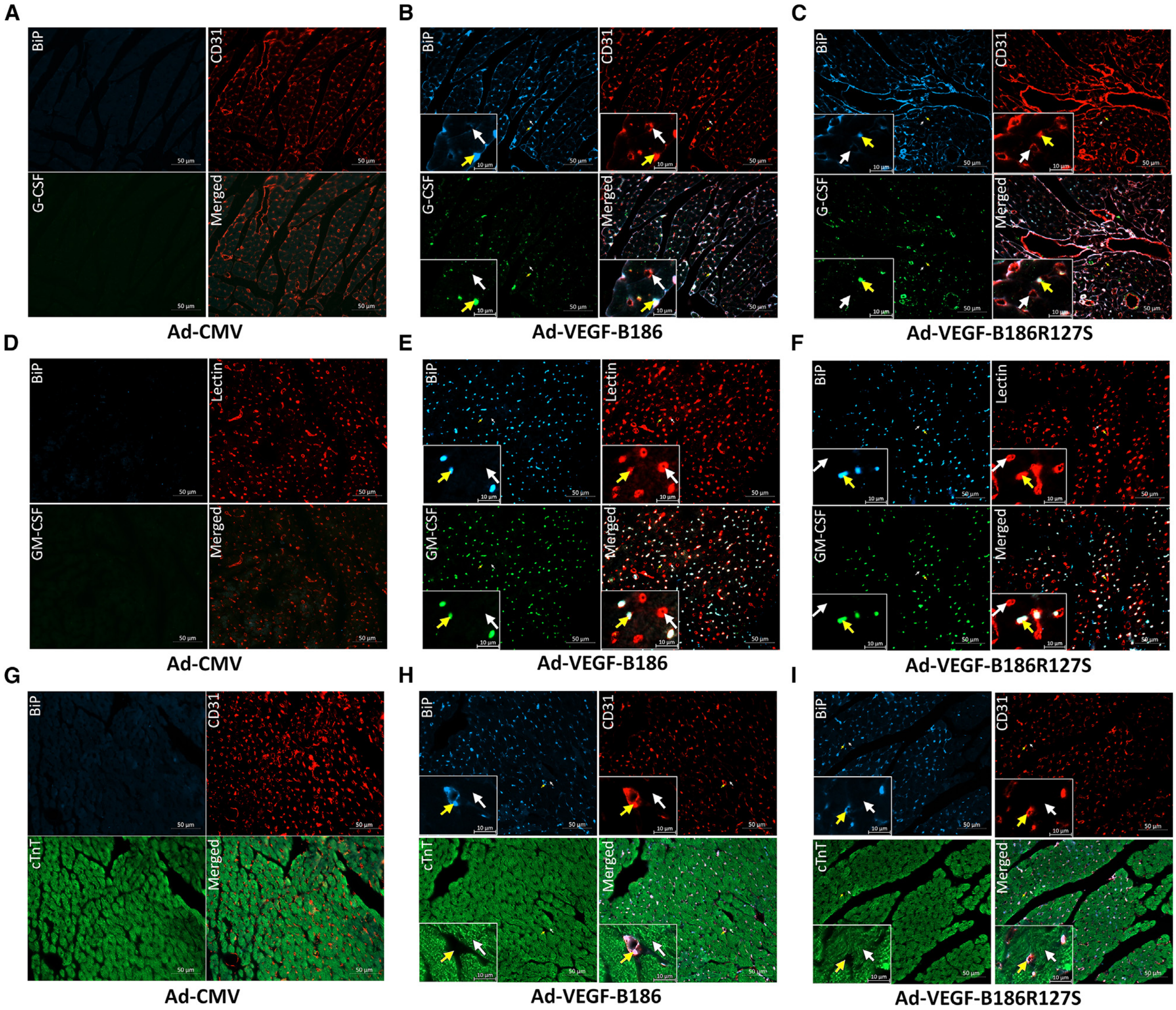

Figure 3. Secretomes with angiogenic potential in the adenoviral vector injected VEGFR1 TK$^{-/-}$ mice hearts

Representative images of immunostained heart tissue sections from VEGFR1 TK$^{-/-}$ mice 6 days after gene transfer. Panels show CD31, G-CSF, and BiP (A–C) (white arrows indicate CD31-stained endothelial cells, while yellow arrows indicate G-CSF-secreting activated endothelial cells); lectin, GM-CSF, and BiP (D–F) (white arrows indicate lectin$^+$ endothelial cells, while yellow arrows indicate lectin$^+$ BiP$^+$ GM-CSF$^+$ activated endothelial cells); and CD31, cTnT, and BiP (G–I) (white arrows indicate cTnT-stained cardiomyocytes, while yellow arrows indicate [BiP and CD31 double-stained] ER-stressed endothelial cells). Scale bars, 50 μm (10 μm for enlarged images).

outcome was observed previously in the presence of VEGFR1.[18] Thus, VEGFR1 signaling is not required for VEGF-B186R127S and VEGF-B186-mediated hematopoietic growth-inducing cytokine secretion. We found a similar outcome at the tissue level. G-CSF and GM-CSF expression was detected in VEGFR1 TK$^{-/-}$ mouse heart sections 6 days following Ad-VEGF-B186R127S, as well as Ad-VEGF-B186 gene transfers (Figures 3A–3F). A similar outcome was observed in wild-type pigs (Figures 4A–4C) and mice (Figures S3A–S3F), as well as in pigs with myocardial infarction (Figures 5A–5F). We also studied mRNA expression of hematopoietic growth-inducing cytokines in cultured HMVEC-Cs. *CSF1, CSF2,* and *CSF3* expressions were significantly upregulated in Ad-VEGF-B186 transduced *FLT1*-depleted HMVEC-Cs (Figures S2D–S2F).

VEGF-B induces ER stress in endothelial cells without VEGFR1 signaling

To explore the mechanisms further, we performed RNA-seq from Ad-VEGF-B186R127S and Ad-VEGF-B186-transduced HMVEC-Cs. Ad-VEGF-B186R127S was shown to induce a similar differential gene expression profile as Ad-VEGF-B186 (Figures 6A, 6B, and S4). Interestingly, the most significantly upregulated genes by adjusted p value (Padj) comprised genes related to endoplasmic reticulum

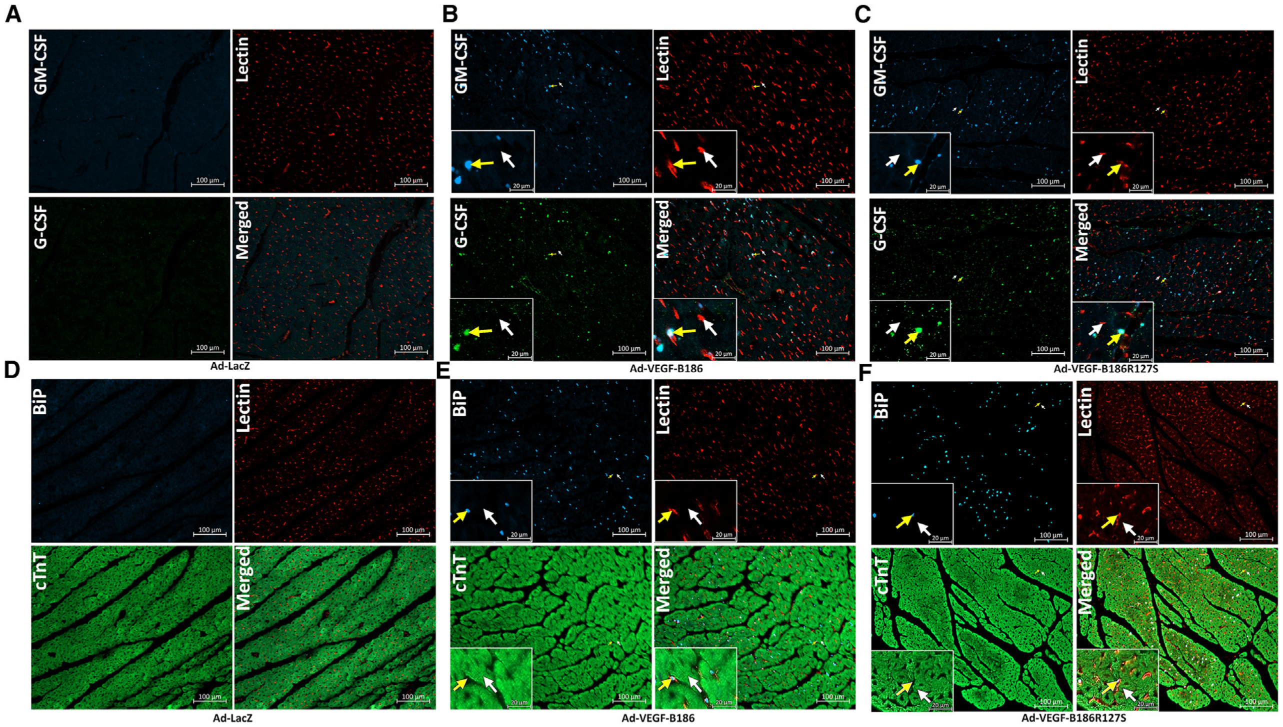

Figure 4. Immunostaining of adenoviral vector transduced porcine hearts

Representative images of triple-immunostained porcine heart tissue sections 6 days after Ad-LacZ, Ad-VEGF-B186, and Ad-VEGF-B186R127S gene transfers. Panels show lectin, G-CSF, and GM-CSF (A–C) (white arrows indicate lectin$^+$ endothelial cells, while yellow arrows indicate G-CSF and GM-CSF-secreting activated endothelial cells) and lectin, cTnT, and BiP (D–F) (white arrows indicate 46 cTnT-stained cardiomyocytes, while yellow arrows indicate [BiP and lectin double-stained] ER-stressed endothelial cells). Scale bars, 100 μm (20 μm for enlarged images).

(ER) stress, which was the most enriched biological process among Ad-VEGF-B186 and Ad-VEGF-B186R127S transduced HMVECs on gene set enrichment analysis (GSEA) (Figures 6A–6D) (Table S3). Furthermore, Ad-VEGF-A165-induced ER-associated gene expression was different with fewer upregulated genes and milder fold-changes than Ad-VEGF-B186 and Ad-VEGF-B186R127S (Figures 6C and 6D) (Table S3).

Cardiac ER stress was also found in VEGFR1 TK$^{-/-}$ mice hearts 6 days following the gene transfers as Ad-VEGF-B186R127S and Ad-VEGF-B186 upregulated ER stress-related protein Binding immunoglobulin Protein (BiP) (Figures 3G–3I). Surprisingly, ER stress was prominent solely in the endothelial cells, but not in cardiomyocytes (Figures 3G–3I). ER stress is known to activate endothelial cells.[24] In this study, the activated endothelial cells produced hematopoietic growth-inducing cytokines, e.g., granulocyte colony-stimulating factor (G-CSF), granulocyte-macrophage colony-stimulating factor (GM-CSF) (Figures 3A–3F). BiP expression was also verified in normoxic (Figures 4D–4F) and ischemic (Figures 5A–5L) pig hearts as well as in wild-type mice (Figures S3A–S3I) following Ad-VEGF-B186R127S and Ad-VEGF-B186 gene transfers. Consistent with the *in vivo* data, we verified that Ad-VEGF-B186 significantly upregulated ER stress-related proteins (BiP, ATF6) as well as G-CSF in HUVECs, which was not seen with Ad-VEGF-A165 (Figures 6E–6H). Similarly, VEGF-B186, but not VEGF-A165, protein treatment was shown to induce ER stress (Figure S5). In HMVEC-Cs, VEGFR1 was dispensable for Ad-VEGF-B186-induced ER stress (Figure S6), as ER stress-related gene expression (*HSPA5*, *ATF6*, *ERN1*, *EIF2AK3*) was similar with or without *FLT1* silencing.

RGD-binding integrins are novel VEGF-B receptors to induce ER stress and angiogenesis

Since VEGFR1 and NRP are not necessary for VEGF-B-induced angiogenesis, it is possible that other so far unrecognized receptors may mediate VEGF-B186 effects. To identify novel receptors of VEGF-B186 contributing to ER stress and angiogenesis, we conducted a crosslinking/MS study to generate a list of VEGF-B186 interacting proteins (Figure S7). Our analysis revealed RGD-binding integrins CD51 (encoded by integrin alpha-V [*ITGAV*]) and CD49e (encoded by *ITGA5*) as novel receptors for VEGF-B186, a finding confirmed by immunoblotting (Table S4; Figures 7A and 7B). We also verified that silencing *FLT1* (Figure 7C) did not have an impact on VEGF-B186 binding to CD51 (ITGAV) or CD49e (ITGA5) (Figure 7D). To elucidate the biological function of RGD-binding integrins, we silenced *ITGAV* and *ITGA5* (Figure S8). Our results demonstrated that RGD-binding integrins, predominantly CD51, serve as key regulators of VEGF-B-mediated ER stress and angiogenic signaling, as *ITGAV* silencing caused downregulation of

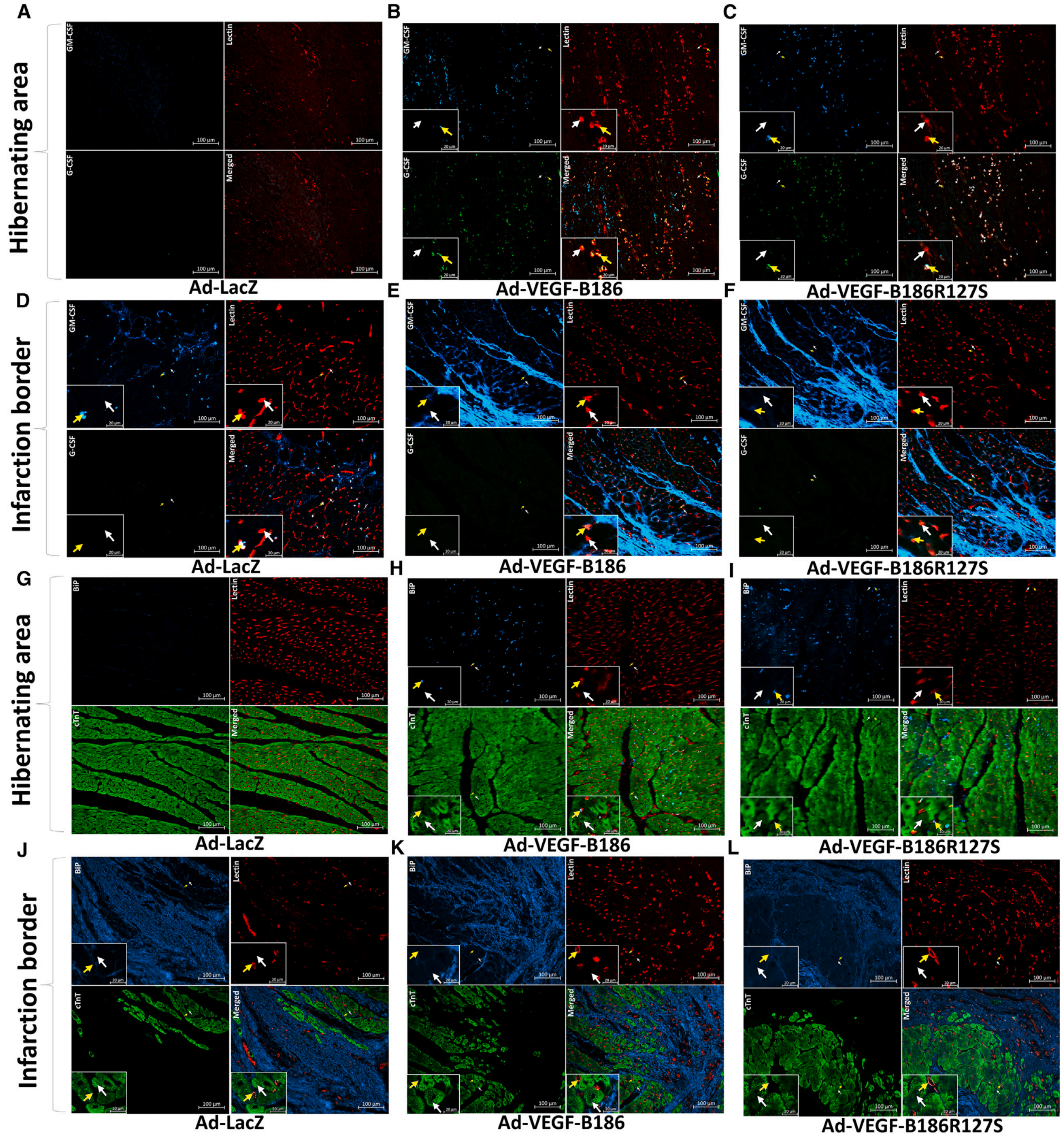

Figure 5. Immunostained adenoviral vector transduced ischemic porcine hearts

(A–C) Representative images of hibernating areas immunostained for lectin, G-CSF, and GM-CSF following Ad-LacZ (A), Ad-VEGF-B186 (B), and Ad-VEGF-B186R127S (C) gene transfers. White arrows indicate lectin-positive endothelial cells; yellow arrows indicate G-CSF/GM-CSF-positive activated endothelial cells. (D–F) Representative images of ischemic border areas immunostained for lectin, G-CSF, and GM-CSF following Ad-LacZ (D), Ad-VEGF-B186 (E), and Ad-VEGF-B186R127S (F) gene transfers. White arrows indicate lectin-positive endothelial cells; yellow arrows indicate GM-CSF-positive activated endothelial cells. (G–I) Representative images of hibernating areas immunostained for lectin, cTnT, and BiP following Ad-LacZ (G), Ad-VEGF-B186 (H), and Ad-VEGF-B186R127S (I) gene transfers. White arrows indicate cTnT-positive cardiomyocytes; yellow arrows indicate endothelial cells double-stained for BiP and lectin. (J–L) Representative images of ischemic border areas immunostained for lectin, cTnT, and BiP following Ad-LacZ (J), Ad-VEGF-B186 (K), and Ad-VEGF-B186R127S (L) gene transfers. White arrows indicate cTnT-positive cardiomyocytes; yellow arrows indicate lectin-positive endothelial cells. Scale bars, 100 μm (20 μm for the enlarged images).

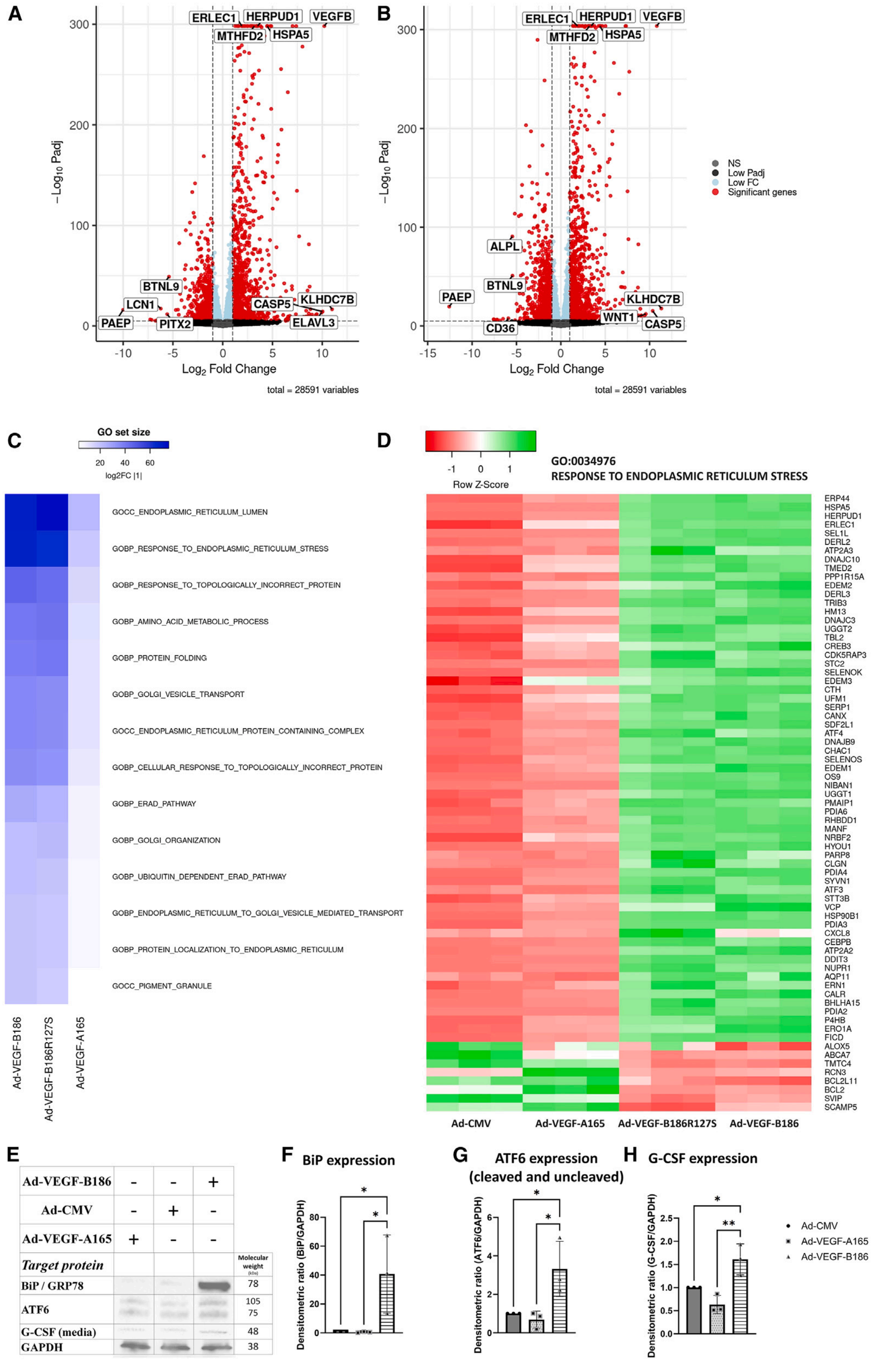

(legend on next page)

ER stress-related gene expression (*ATF6*, *ERN1*, *EIF2AK3*), proangiogenic gene expression (*VEGF-A* and *ANGPT2*), as well as hematopoietic growth-inducing cytokine gene expression (*CSF1, CSF2,* and *CSF3*) (Figures 8A–8H).

XBP1 regulates VEGF-B-mediated activation of endothelial cells to induce angiogenesis

As the ER stress-related protein BiP along with the hematopoietic growth-inducing cytokines G-CSF and GM-CSF were co-expressed in cardiac endothelial cells (Figure 3), we sought to determine whether VEGF-B-induced ER stress provokes angiogenesis in endothelial cells or if the two effects are independent. To investigate this, we silenced *XBP1*, a transcription factor containing a bZIP domain associated with ER stress (Figure S9A).[25,26] We observed a significant reduction in VEGF-B186-mediated proangiogenic and hematopoietic growth-inducing cytokine gene expression (Figures 8I–8M). Additionally, *XBP1* silencing led to a significant reduction in the expression of the ER stress chaperone BiP (Figures S9B and S9C).

DISCUSSION

The role of VEGF-B in cardiac function has been a topic of ongoing investigation, with previous studies providing insights into its angiogenic and non-angiogenic effects.[14,27,28] Unlike VEGF-A, VEGF-B has been shown to exert a limited angiogenic effect while predominantly stimulating non-endothelial cells in the heart, particularly mesenchymal stromal cells,[13,29] indicating a unique mechanism of action in cardiac remodeling. Further studies are needed to characterize the proliferating mesenchymal stromal cells more thoroughly. Building upon this understanding, our study demonstrates that VEGF-B-mediated cellular proliferation in the heart is independent of VEGFR1 and NRP1/2 signaling pathways, offering further insights into its function in cardiac tissue.

A new mechanistic aspect of the current study is the recognition of the VEGF-B impact on ER stress, a crucial regulator of cardiovascular function.[30] We demonstrate that VEGF-B induces distinct ER stress responses compared with VEGF-A165, implicating it as a potential modulator of ER homeostasis in cardiac microvascular endothelial cells. Given the link between ER stress and cardiovascular pathology, our findings underscore the significance of understanding the dual role of the UPR in either protecting or exacerbating cardiovascular conditions.[30,31]

Our study revealed that VEGF-B induces ER stress independently of the VEGFR1 downstream signaling, acting via RGD-binding integrins. Integrins play crucial roles as regulators of cell survival, proliferation, adhesion, and migration. Upon activation, integrins facilitate controlled interactions between the extracellular factors and the cytoskeleton.[32] The inflammatory and angiogenic roles, as well as the role in ER stress of the RGD-binding integrins have been well-established in previous studies.[33–37] We have recently shown that the angiogenic potential of these VEGF-B isoforms is at least partly due to the recruitment of endothelial progenitor cells.[18] A recent study by Sultan and colleagues also confirmed the expansion of the induced endothelial cell population in VEGF-B transgenic mice.[38]

Furthermore, our study highlights the interplay between VEGF-B-induced ER stress and angiogenesis through the upregulation of hematopoietic growth-inducing cytokines in endothelial cells. These cytokines play a pivotal role in recruiting endothelial progenitor cells and attenuating inflammation,[39–43] thus providing a mechanistic link among VEGF-B, ER stress, and angiogenesis. Additionally, we demonstrate here that VEGF-B186 gene therapy upregulates ER stress sensors (ATF6, IRE1α, and PERK) and UPR-associated proangiogenic genes like *VEGFA* and *ANGPT2*,[44,45] further elucidating the molecular mechanisms underlying VEGF-B-mediated effects.

Our study also shows the role of XBP1, a key transcription factor in the UPR, in regulating VEGF-B-mediated endothelial activation. The expression of BiP is increased when XBP1 interacts with the UPR element in BiP's promoter region.[46] ATF6 upregulation induces *XBP1* mRNA expression while IRE1α causes splicing of *XBP1* mRNA.[46] XBP1 has the potential to contribute to angiogenic signaling,[47–49] suggesting that XBP1 at least partially regulates VEGF-B-induced angiogenesis. This provides additional insight into the intricate pathways involved in the effects of VEGF-B on cardiac tissue.

We identified the upregulation of the ER chaperone BiP exclusively in endothelial cells following VEGF-B gene therapy, underscoring its role in mediating VEGF-B-induced effects on endothelial activation, angiogenesis, and inflammation.[24,50,51] RNA sequencing results suggest a potential mechanism for mitigating VEGF-B-induced ER stress through the upregulation of MANF expression,[52–54] further highlighting the intricate balance between ER stress and cellular homeostasis.

However, it is essential to acknowledge the potential long-term adverse effects of VEGF-B-induced ER stress, including cardiac dysfunction and dilated cardiomyopathy,[55] which warrant caution in considering long-term VEGF-B expression as a therapeutic strategy for heart diseases. In certain conditions VEGF-B overexpression can even lead to cardiac hypertrophy.[38,56] Also, the risk of VEGF-B-induced arrhythmias increases if VEGF-B186R127S is not used, as the cleaved C-terminal end of VEGF-B186 is found to be pro-arrhythmogenic.[17]

Figure 6. ER stress-related gene expression in adenoviral vector transduced HMVEC-Cs and protein expression in HUVECs

Volcano plots showing significantly altered genes in HMVEC-Cs treated with Ad-VEGF-B186 (A) and Ad-VEGF-B186R127S (B). GO term heatmap (C) summarizes RNA-seq data. Genes involved in ER stress are shown in (D). Significant genes = Padj <0.05, log2FC|1| or greater. Immunoblots (E) and quantifications (F–H) of BiP/GRP78, ATF6, and G-CSF expression in HUVECs transduced with Ad-VEGF-B186, Ad-VEGF-A165, and Ad-CMV (n = 3). Horizontal bars indicate mean ± SD and p values vs. each group by one-way ANOVA, followed by Dunnett's multiple comparison test (* $p < 0.05$, ** $p < 0.005$).

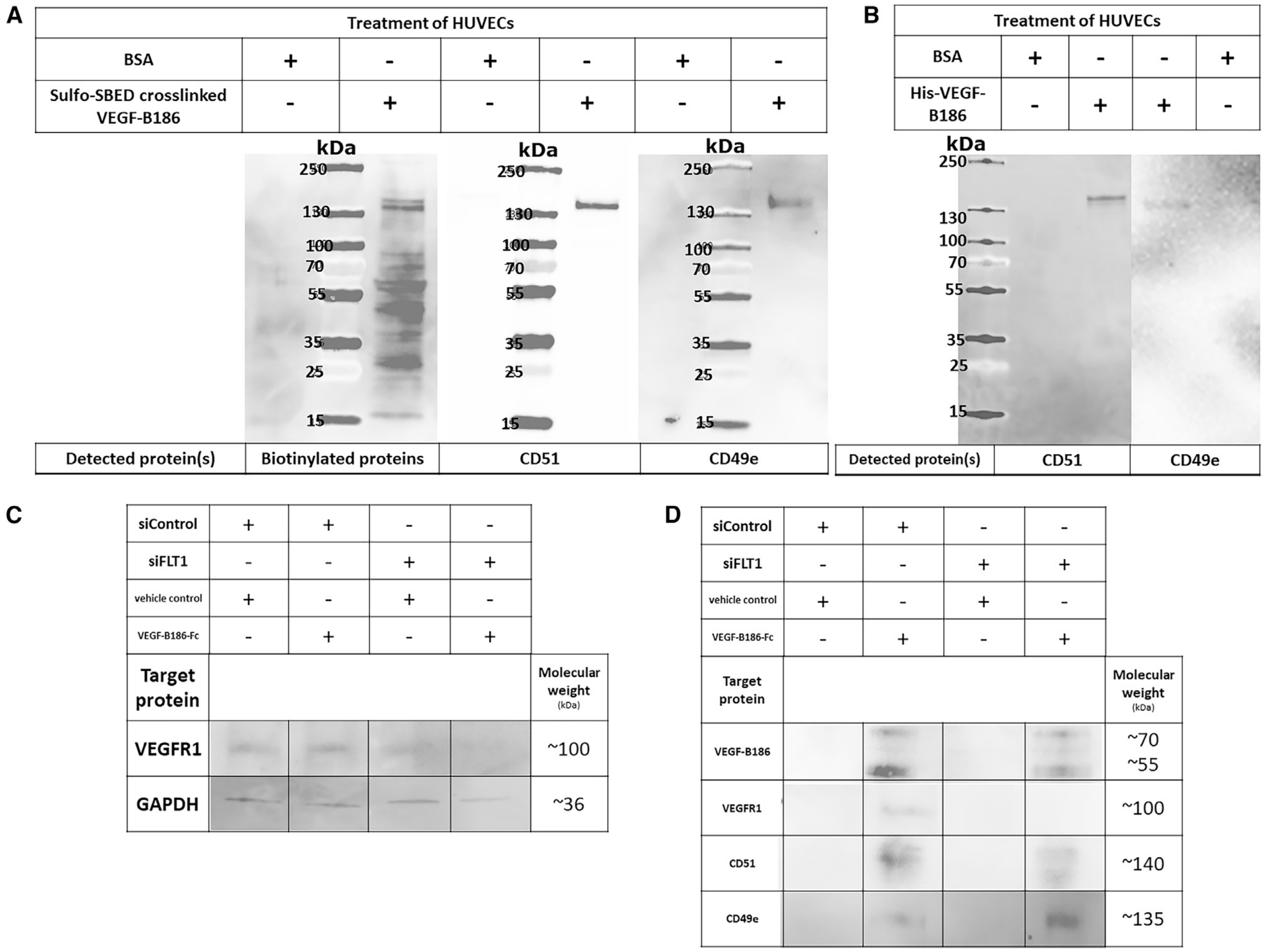

Figure 7. The RGD-binding integrins are novel receptors for VEGF-B186

(A) Immunoblot of biotin-labeled proteins and RGD-binding integrins CD51 (ITGAV) and CD49e (ITGA5) from VEGF-B186-treated HUVEC lysates after sulfo-SBED crosslinking. (B) Immunoblot of CD51 and CD49e detected in complexes pulled down with His-tagged VEGF-B186 from HUVECs. (C) Immunoblot of VEGFR1 expression in siFLT1-treated HUVECs before VEGF-B186-Fc pull-down. (D) Immunoblot of proteins (VEGF-B, VEGFR1, CD51, and CD49e) co-precipitated with VEGF-B186-Fc from siFLT1-treated HUVECs.

In conclusion, our study provides novel insights into the mechanisms underlying VEGF-B-mediated effects in the heart, emphasizing the potential of VEGF-B186R127S gene therapy as a promising therapeutic candidate for the treatment of cardiac diseases.

MATERIALS AND METHODS

Ultrasound-guided closed-chest intramyocardial injections into mouse models

A total of 15 VEGFR1 tyrosine kinase domain knockout (VEGFR1 $TK^{-/-}$) C57BL/6JOlaHsd male mice aged 18 weeks on a standard chow diet were used for the experiments. VEGFR1 $TK^{-/-}$ mice are ablated of VEGFR1 downstream signaling.[57] Also, a total of 19 specific pathogen-free wild-type C57BL/6J male mice aged 18 weeks under a standard chow diet were used for the experiments. Mice were kept under 12 light/12 dark cycles, temperatures of 22 ± 2°C with 50% ± 10% humidity in The National Laboratory Animal Center of The University of Eastern Finland, Kuopio, Finland. All animal procedures were approved by The National Animal Experimental Board of Finland and carried out by the guidelines of The Finnish Act on Animal Experimentation. The study was conducted in strict accordance with the guidelines outlined in Directive 2010/63/EU of the European Parliament on the protection of animals used for scientific purposes. Ultrasound-guided closed-chest intramyocardial adenoviral vector injections (final concentration of 1×10^{12} viral particles [vp]/mL, and a total of 1×10^{10} vp in 10 μL) were done under anesthesia.[18] Anesthesia was achieved by inhalation of isoflurane (1%–4%) and monitored by respiratory rate and withdrawal reflex. Animals were euthanized by CO_2 inhalation. Study groups are described in Table S1.

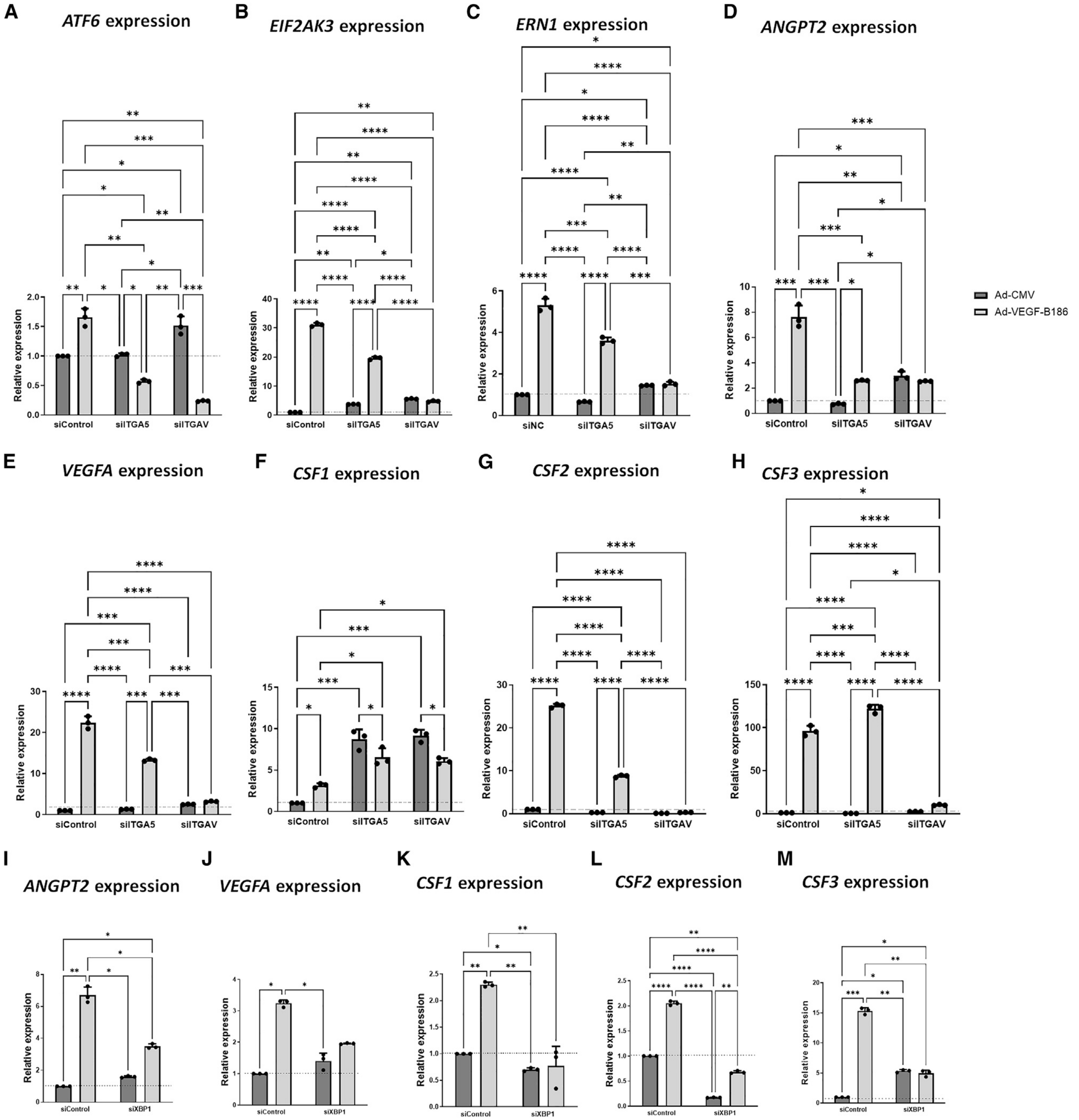

Figure 8. Regulation of VEGF-B186-induced ER stress and angiogenesis related signaling

RNA expression of ER stress-related and angiogenic genes (A–H) in siControl (n = 3), siITGA5 (n = 3), or siITGAV (n = 3)-treated TeloHAECs after Ad-VEGF-B186 transduction. RNA expression of *ANGPT2* (I), *VEGFA* (J), *CSF1* (K), *CSF2* (L), and *CSF3* (M) in siControl (n = 3) or siXBP1 (n = 3)-treated HUVECs after Ad-VEGF-B186 transduction. Horizontal bars indicate mean ± SD and p values vs. each group by two-way ANOVA followed by Tukey's multiple comparison test. The n value represents the number of individual cell culture experiments, which are defined as biological replicates. *$p < 0.05$, **$p < 0.005$, ***$p < 0.0005$, ****$p < 0.0001$.

Intramyocardial injections into porcine models

A total of 33 female domestic pigs, aged 3 months and on a standard chow diet, were used for the experiments. Eighteen pigs were used to compare the effects of Ad-VEGF-B isoforms in healthy porcine myocardium. To perform gene transfers to ischemic myocardium, ischemia was induced in another 15 pigs by placing a bottleneck stent

in the LAD 2 weeks before the gene transfer into the hibernating myocardial area.[58] All animal procedures were carried out in accordance with the ARRIVE guidelines and the UK Animals Act and were authorized by the Animal Experiment Board in Finland. The study was conducted in strict accordance with the guidelines outlined in Directive 2010/63/EU of the European Parliament on the protection of animals used for scientific purposes. Prior to the procedures, pigs were sedated with an intramuscular injection of 1.5 mL atropine and 6 mL azaperone. Following this initial sedation, general anesthesia was administered using propofol at a dose of 15 mg/kg/h and fentanyl at 10 μg/kg/h. The MyoStar intramyocardial injection catheter (Johnson & Johnson, California, USA) was inserted into the left ventricle through the femoral sheath under fluoroscopic guidance (GE Innova 3100IQ 3D, GE Healthcare, Waukesha, WI) on day 0 of the experiments. Ten 0.2-mL injections of adenoviral product were administered to the anterolateral wall of the left ventricle, with each heart receiving a total of 1×10^{12} vp.[17] The animals were euthanized with an intravenous injection of KCl while under general anesthesia. Study groups are described in Table S1.

Viral constructs

Adenoviral vectors encoding VEGF-B186 and proteolytically resistant VEGF-B186R127S were produced by the Biocenter Kuopio National Virus Vector Laboratory under GMP-like conditions.[18,59,60] All constructs, including empty control virus, carry cytomegalovirus (CMV) promoter. The constructs have previously been characterized.[18]

Cell culture

Human cardiac microvascular endothelial cells (HMVEC-Cs) (Lonza, CC-7030), human umbilical vein endothelial cells (HUVECs), and immortalized human aortic endothelial cells (TeloHAECs) (ATCC, CRL-4052) were used for adenoviral vector transductions. HMVEC-Cs were cultured according to the manufacturer's protocol (Lonza, USA) and steps have been described by Mallick et al.[18] The study involving HUVECs was conducted in accordance with the ethical principles outlined in the Declaration of Helsinki. All procedures were reviewed and approved by the ethical committee of the Kuopio University Hospital. Informed written consent was obtained from all donors prior to the inclusion of umbilical cords in the study. Isolated HUVECs from umbilical cords were cultured in Endothelial Cell Growth Medium (Promocell, C-22010-500ML) on fibronectin-gelatin-coated surfaces.[61] And TeloHAECs were cultured in Vascular Cell Basal Medium (ATCC, PCS-100-030) according to the manufacturer's protocol.

Protein treatments and crosslinking protein interaction analysis

Cultured HUVECs were treated with 500 ng of VEGF-B186 (ProSci Incorporated, 96–775), or 500 ng of VEGF-A165 (R&D systems; BT-VEGF-GMP), or 1% BSA (control) for 2 h and 16 h. Media and cell lysates were then collected and normalized using Pierce BCA Protein Assay Kits (Thermo Scientific, 23225). The crosslinking study experiments were performed using Sulfo-SBED Biotin Label Transfer Reagent (Thermo Scientific, 33033).[62] A total of 2 μg of VEGF-B186 (ProSci Incorporated, 96–775) was incubated with 50 ng/μL of sulfo-SBED in 1 mL PBS at 4°C for 2 h with gentle agitation. The nonreacted free sulfo-SBED was inactivated by the addition of 50 mM Tris-HCl (pH 7.5). The confluently cultured HUVECs or TeloHAECs were incubated with sulfo-SBED-labeled VEGF-B186 (2 μg in 1 mL of HEPES buffer) at 4°C for 1 h with gentle agitation. The mixture was then exposed to UV light, 312 nm at room temperature (RT). Stepwise, cells were incubated with 200 mM DTT (37 mg DTT in 1 mL PBS) at 4°C for 1 h before lysing the cells for the immunoblotting experiment.

Pull-down assay

Pull-down experiments were performed using either Ni-NTA magnetic beads (Thermo Scientific, 88831) or Pierce protein A/G magnetic beads (Thermo Scientific, 88803). For the pull-down of His-VEGF-B186 (ProSci Incorporated, 96–775), first 8.8×10^6 HUVECs were seeded in 10-cm dishes and treated with 5 μg of His-VEGF-B186 or 1% BSA (control) for 1 h at 4°C. Cells were then lysed using Pierce IP Lysis Buffer (Thermo Scientific, 87787). Similarly, for the pull-down assay of VEGF-B186-Fc (Sino biological, 96–775), HUVECs were treated with either 5 μg VEGF-B186-Fc or 1% BSA on six-well dishes for 1 h at 4°C before cell lysis. The lysates were centrifuged at 3,500 rpm for 10 min at 4°C to remove debris. The resulting supernatants were incubated with either Ni-NTA beads or protein A/G magnetic beads at 4°C for 2 h. After incubation, the beads were washed three times with washing buffer. Bound proteins were eluted by adding 4× Laemmli buffer (for Ni-NTA beads) or Pierce immunoglobulin (Ig)G elution buffer (Thermo Scientific, 21004) (for protein A/G magnetic beads). Following neutralization of the IgG elution buffer and addition of 4× Laemmli buffer, the eluates were incubated for 5 min at 96°C. The eluted proteins were then resolved by SDS-PAGE and subjected to immunoblotting analysis.

siRNA transfection

Cultured cells were transfected with the indicated dicer-substrate small interfering RNAs (siRNAs) targeting human *FLT1*, *XBP1*, *ITGA5*, *ITGAV* (Integrated DNA Technologies) or a non-targeting negative control siRNA (Integrated DNA Technologies, 11-01-03-01). Transfection of siRNAs into cultured cells was performed using Oligofectamine Transfection Reagent (Invitrogen, 12252011) according to the manufacturer's protocol. Briefly, 50 nM of siRNA was used, and knockdown was assessed by real-time PCR. Viral vector transductions were conducted 24 h after the siRNA transfections.

Adenoviral vector transduction

Following the seeding of HMVEC-Cs, HUVECs, or TeloHAECs into six-well plates, cells were incubated overnight in a humidified atmosphere with 5% CO_2 at 37°C in the corresponding cell line growth medium. The next day, cells were transduced with 1,000 vp/cell. Twenty-four hours later, cells were washed with PBS and a fresh cell growth medium was added.

RNA extraction and sequencing

RNA from cultured cells was extracted with RNeasy mini kit (Qiagen, 74106) according to the manufacturer's protocol. RNA library preparation and sequencing were performed by Genewiz (Azenta Life

Sciences, Germany). Briefly, unstranded sequencing libraries were constructed using NEBNext Ultra II RNA Library Preparation Kit with polyA-selection from three biological replicates of each treatment (Ad-VEGF-B186, Ad-VEGF-B186R127S, Ad-VEGF-A165, and Ad-CMV) and paired-end sequencing was performed with Illumina NovaSeq6000.

RNA-seq data analysis and visualization

Data preprocessing was done by Genewiz. Sequencing reads were trimmed with Trimmomatic (v.0.36) and aligned to GRCh38 human reference genome (ENSEMBL) using STAR (v.2.5.2b).[63,64] Raw gene count table was retrieved with featureCounts from the Subread package (c1.5.2).[65]

Differential gene expression (DGE) analysis and further data visualization were done with R programming language (v.4.2.2). The DGE analyses between targets (Ad-VEGF-B186, Ad-VEGF-B186R127S. Ad-VEGF-A165) and control (Ad-CMV) were conducted with DESeq2 (v.1.38.3) and apeglm (v.1.20.0) fold change (FC) shrinkage following standard workflow using the Wald test for statistics.[66,67] Low-expressing genes (<5 counts in total among all samples) were filtered away from the count matrix before DGE analysis. Genes with adjusted p value (Padj) < 0.05 and FC > 1 were considered significant. Ensembl ID:s were converted to symbols using gConvert from gprofiler2 (v.0.2.2) and volcano plots were generated using EnhancedVolcano (v.1.16.0) in which the top four genes were labeled according to the lowest Padj, and the greatest down- and upregulation by FC.[68] A PCA plot was generated from VST counts with default settings of DESeq2 as in the standard workflow. A Venn diagram of significantly altered genes was constructed using VennDiagram (v.1.7.3). All genes of the DESeq2 result tables were raked in before gene set enrichment analysis (GSEA) by FC-signed logarithmic Padj (−log10(Padj)/FCsign). GSEAs were performed for treatment vs. control DGE analysis results with GSEABase (v.1.60.0) and clusterProfiler (v.4.6.2) using MSigDB human gene ontology (GO) reference (C5 GO Hs. symbols, v.2023.1).[69–72] The most significant gene sets and an individual GO set were visualized with Enhanced Heatmaps by gplots (v.3.1.3). GSEA results were ranked individually according to GO terms q-value, and significant genes associated with the top 10 gene sets of each treatment were counted and plotted into a heatmap. The most significant biological processes were visualized using DESeq2 normalized gene counts filtered with the GO-associated, significantly altered genes. Colors for the Venn diagram and the heatmaps were retrieved using RColorBrewer (v.1.1–3).

Quantitative real-time PCR analyses

One microgram of total RNA from cultured cells was reverse transcribed into cDNA using random hexamers and RevertAID reverse transcriptase (Thermo Fisher Scientific, EP0441). Quantitative real-time PCR was performed using Powerup SYBR Green Master mix (Applied Biosystems, A25741) and QuantStudio3 (Applied Biosystems) with the indicated primers (Table S2). The real-time PCR data were analyzed with QuantStudio Software (Applied Biosystems). Results were calculated using the delta-delta CT method ($2^{-\Delta\Delta CT}$)[73] and normalized with *GAPDH*.

Immunoblotting

Protein samples were fractionated by SDS-polyacrylamide gel electrophoresis and blotted onto nitrocellulose membranes (Bio-Rad, 1704159) and blocked for 1 h with 5% BSA (bovine serum albumin) in TBST (0.1% Tween 20 in TBS). Anti-GRP78 (Invitrogen, MA5-15619), anti-ATF6 (Novus Biologicals, NBP1-40256SS), anti-human G-CSF (Novus Biologicals, NBP2-52447), anti-ITGAV (Invitrogen, MA5-32195), anti-ITGA5 (Invitrogen, PA5-96530), anti-streptavidin (Vector Laboratories, SA-5014-1), anti-XBP1s (Cell Signaling Technology, 40435) and anti-GAPDH (R&D systems, MAB5718) primary antibodies were incubated overnight at 4°C. After washing, the membranes were treated for 1 h at RT with secondary peroxidase-linked antibodies (anti-mouse [R&D systems, HAF018] and anti-rabbit [Invitrogen, 31460]). ECL western blot detection solution (Thermo Scientific, 32132) was used to detect target proteins.

On membrane digestion and preparation for mass spectrometry

Areas of interest in the nitrocellulose membrane were cut out and immersed in milliQ water. Subsequently, the cut membranes were treated with Solution P (0.5% [w/v] polyvinylpyrrolidone in 100 mM acetic acid) (Sigma-Aldrich; 9003-39-8) at 37°C, washed, and cut into small pieces. These pieces were then incubated with Tris hydrochloride buffer (pH 8.5) (MP Biomedicals; 1185-53-1), and the proteins on the membrane were reduced by 50 μg of DTT for 1 h followed by alkylation using 125 μg of iodoacetamide (Sigma-Aldrich; 144-48-9) for 1 h in the dark while mixing. The membrane pieces containing reduced and alkylated proteins were then treated with Solution D (10% acetonitrile in 20 mM Tris-HCl, pH 9.0). The proteins were then digested with 0.05% ProteaseMAX Surfactant (Promega; V2071), and 0.5 μg of Lysyl Endopeptidase, (Promega; V1671) at 30°C for 3 h, and then with 1 μg of modified trypsin (Promega; V5113) at 37°C for 16 h. The digested tryptic peptides were desalted using GL-Tip SDB (GL Sciences; 7820–11200) and GC (GL Sciences; 7820–11201) according to the manufacturer's protocol, and vacuum dried. The samples were dissolved in 50 μL of 2% acetonitrile acidified with 0.1% formic acid prior to liquid-chromatography/tandem mass spectrometry (LC-MS/MS) analysis.

LC-MS/MS protein analysis

Proteomics analysis was conducted using UPLC (Vanquish Flex, Thermo Scientific) coupled to an Orbitrap Q Exactive mass spectrometer in positive ion mode. Peptides were separated on an Agilent AdvanceBio Peptide Map column over an 80-min gradient from 2% to 45% buffer B. MS detection was conducted by following data independent acquisition mode as previously described.[74] Data were processed by DIA-NN software,[75] with the UniProt proteome ID: UP000005640; taxon ID: 9606, applying 1% false discovery rate thresholds. MaxLFQ normalized intensities were used for data evaluation.

Multiplex protein level measurement

Protein levels (VEGF-A, Angiopoietin-2) from murine plasma were analyzed on a single Luminex platform (R&D systems, LXSAMSM-08) according to the manufacturer's instructions as described.[18]

Histological analyses

Perfused mouse and pig hearts were embedded in paraffin and cut into 5- to 6-μm sections, which were used for histological analyses.[18] Microvascular areas (%) were measured under a Nikon H550L microscope from CD31 (1:200, BD Pharmingen, 550274) immunostained sections at 40× magnification. Fiji ImageJ2 software was used in a blinded manner from four different fields of randomly selected tissue sections to measure the microvascular areas.

For immunofluorescence studies, harvested tissues were deparaffinized and rehydrated, followed by blocking with 5% bovine serum albumin (Sigma-Aldrich, 9048-46-8) in permeabilization solution (0.25% Triton X-100 in PBS) overnight. Then sectioned tissues were incubated for 1 h at RT with the following primary antibodies (diluted at a ratio of 1:200 in blocking solution): anti-VEGF-B (Invitrogen, MA5-26326), anti-cardiac troponin T (TnT) (Invitrogen, MA5-12960), anti-CD31 (Cell signaling, 77699), biotinylated GSL I (Vector Laboratories, B-1105-2), anti-laminin (Abcam, ab11575), anti-BiP (Invitrogen, MA5-15619), anti-G-CSF (OriGene, TA375138), anti-mouse GM-CSF (eBioscience, 14-7331-85), anti-human GM-CSF (R&D Systems, MAB215), anti-PDGFR-β (Abcam, ab32570), anti-c-kit (R&D systems, AF1356), and anti-Ki67 (eFlour 570 conjugated) (Invitrogen, 41-5698-82). After several washes with PBST (0.025% Triton X-100 in PBS), the samples were incubated with the following secondary antibodies diluted at a ratio of 1:500 in PBS for 30 min: A350 goat anti-mouse secondary antibody (Invitrogen, A-21050), A488 goat anti-rabbit secondary antibody (Invitrogen, A-11008), A488 chicken anti-rabbit secondary antibody (Invitrogen, A-21441), A488 chicken anti-rat secondary antibody (Invitrogen, A-21470), and A594 chicken anti-rabbit secondary antibody (Invitrogen, A-21442). Biotinylated lectin was detected by incubating with Texas red avidin D (Vector Laboratories, A-2006-5). Mounting was performed with either antifade vectamount AQ medium (Vector Laboratories, H-5501-60) or antifade Vectashield mounting medium with DAPI (Vector laboratories, H-1200) before fluorescence imaging using ZEISS Axio Imager 2 microscope. Fiji ImageJ2 software was used in a blinded manner from four different fields of randomly selected immunostained tissue sections to count the proliferating cell numbers.

Statistics and reproducibility

Data presentations were done as mean ± standard deviation (SD). Statistical differences between the means were compared by the two-tailed, unpaired t test for two groups, or determined using one-way ANOVA followed by Dunnett's multiple comparison test or two-way ANOVA followed by Šidák's test for multiple groups. Statistical analysis was performed with Prism version 9 (GraphPad Software). Statistical significance was set to p value <0.05 (p value style: *<0.05, **<0.005, ***<0.0005, ****<0.0001). Non-significant p values were not mentioned.

DATA AVAILABILITY

The datasets used and/or analyzed during the current study are available from the corresponding author on reasonable request.

ACKNOWLEDGMENTS

We thank Tiina Koponen and Sari Järveläinen for their work on adenoviral vector production at the Biocenter Kuopio National Virus Vector Laboratory. We also thank Kanako Niitsu for preparing the samples for mass spectrometry. Additionally, we acknowledge the personnel of the Laboratory Animal Center for their care of the animals. This study was supported by Research Council of Finland Flagship Project GeneCellNano (337120) (to S.Y.-H.), ERC Advanced Grant (GA884382) (to S.Y.-H.), Research Council of Finland (339560) (to P.K.), Finnish Foundation for Cardiovascular Research (230057) (to R.M.), Finnish Cultural Foundation (65221677) (to R.M.), Antti and Tyyne Soininen Foundation (to R.M.), Orion Research Foundation (to R.M.), and Aarne Koskelo Foundation (to R.M.).

AUTHOR CONTRIBUTIONS

R.M. and T.N. designed all the experiments. R.M. and M.B. conducted *in vitro* experiments. R.M., A.B.M., and T.T. contributed to the crosslinking protein interaction experiment. R.M., A.T., and E.G. contributed to echocardiography-guided intramyocardial injections in mice models, blood collection, and analyses of echocardiographic data. H.K. contributed to intramyocardial injections in porcine models and collected hearts for histological analysis. G.J. and P.K. analyzed RNA sequencing data. R.M. performed histological analyses, RT-PCR, and immunocytochemistry of mice samples. R.M. also analyzed and curated all the data. R.M., A.B.M., G.J., and T.N. wrote the manuscript. T.N. and S.Y.-H. designed the study and conducted supervision, editing, and reviewing of the manuscript. S.Y.-H. conducted funding acquisition.

DECLARATION OF INTERESTS

The authors declare that the research was conducted in the absence of any commercial or financial relationships that could be construed as a potential conflict of interest.

SUPPLEMENTAL INFORMATION

Supplemental information can be found online at https://doi.org/10.1016/j.ymthe.2025.03.012.

REFERENCES

1. Ylä-Herttuala, S., and Alitalo, K. (2003). Gene transfer as a tool to induce therapeutic vascular growth. Nat. Med. *9*, 694–701. https://doi.org/10.1038/nm0603-694.
2. Ralapanawa, U., and Sivakanesan, R. (2021). Epidemiology and the Magnitude of Coronary Artery Disease and Acute Coronary Syndrome: A Narrative Review. J. Epidemiol. Glob. Health *11*, 169–177. https://doi.org/10.2991/jegh.k.201217.001.
3. Bergmann, O., Bhardwaj, R.D., Bernard, S., Zdunek, S., Barnabé-Heider, F., Walsh, S., Zupicich, J., Alkass, K., Buchholz, B.A., Druid, H., et al. (2009). Evidence for Cardiomyocyte Renewal in Humans. Science *324*, 98–102. https://doi.org/10.1126/science.1164680.
4. Ylä-Herttuala, S., Bridges, C., Katz, M.G., and Korpisalo, P. (2017). Angiogenic gene therapy in cardiovascular diseases: dream or vision? Eur. Heart J. *38*, 1365–1371. https://doi.org/10.1093/eurheartj/ehw547.
5. Kloner, R.A., King, K.S., and Harrington, M.G. (2018). No-reflow phenomenon in the heart and brain. Am. J. Physiol. Heart Circ. Physiol. *315*, H550–H562. https://doi.org/10.1152/ajpheart.00183.2018.
6. Risau, W., and Flamme, I. (1995). Vasculogenesis. Annu. Rev. Cell Dev. Biol. *11*, 73–91. https://doi.org/10.1146/annurev.cb.11.110195.000445.
7. Olofsson, B., Korpelainen, E., Pepper, M.S., Mandriota, S.J., Aase, K., Kumar, V., Gunji, Y., Jeltsch, M.M., Shibuya, M., Alitalo, K., and Eriksson, U. (1998). Vascular endothelial growth factor B (VEGF-B) binds to VEGF receptor-1 and regulates plasminogen activator activity in endothelial cells. Proc. Natl. Acad. Sci. USA *95*, 11709–11714. https://doi.org/10.1073/pnas.95.20.11709.
8. Clauss, M., Weich, H., Breier, G., Knies, U., Röckl, W., Waltenberger, J., and Risau, W. (1996). The Vascular Endothelial Growth Factor Receptor Flt-1 Mediates Biological Activities. J. Biol. Chem. *271*, 17629–17634. https://doi.org/10.1074/jbc.271.30.17629.
9. Katz, T.C., Singh, M.K., Degenhardt, K., Rivera-Feliciano, J., Johnson, R.L., Epstein, J.A., and Tabin, C.J. (2012). Distinct Compartments of the Proepicardial Organ Give

Rise to Coronary Vascular Endothelial Cells. Dev. Cell *22*, 639–650. https://doi.org/10.1016/j.devcel.2012.01.012.

10. Chen, H.I., Sharma, B., Akerberg, B.N., Numi, H.J., Kivelä, R., Saharinen, P., Aghajanian, H., McKay, A.S., Bogard, P.E., Chang, A.H., et al. (2014). The sinus venosus contributes to coronary vasculature through VEGFC-stimulated angiogenesis. Development *141*, 4500–4512. https://doi.org/10.1242/dev.113639.

11. Wu, B., Zhang, Z., Lui, W., Chen, X., Wang, Y., Chamberlain, A.A., Moreno-Rodriguez, R.A., Markwald, R.R., O'Rourke, B.P., Sharp, D.J., et al. (2012). Endocardial Cells Form the Coronary Arteries by Angiogenesis through Myocardial-Endocardial VEGF Signaling. Cell *151*, 1083–1096. https://doi.org/10.1016/j.cell.2012.10.023.

12. Red-Horse, K., Ueno, H., Weissman, I.L., and Krasnow, M.A. (2010). Coronary arteries form by developmental reprogramming of venous cells. Nature *464*, 549–553. https://doi.org/10.1038/nature08873.

13. Lähteenvuo, J.E., Lähteenvuo, M.T., Kivelä, A., Rosenlew, C., Falkevall, A., Klar, J., Heikura, T., Rissanen, T.T., Vähäkangas, E., Korpisalo, P., et al. (2009). Vascular Endothelial Growth Factor-B Induces Myocardium-Specific Angiogenesis and Arteriogenesis via Vascular Endothelial Growth Factor Receptor-1– and Neuropilin Receptor-1–Dependent Mechanisms. Circulation *119*, 845–856. https://doi.org/10.1161/CIRCULATIONAHA.108.816454.

14. Bry, M., Kivelä, R., Leppänen, V.-M., and Alitalo, K. (2014). Vascular Endothelial Growth Factor-B in Physiology and Disease. Physiol. Rev. *94*, 779–794. https://doi.org/10.1152/physrev.00028.2013.

15. Huusko, J., Lottonen, L., Merentie, M., Gurzeler, E., Anisimov, A., Miyanohara, A., Alitalo, K., Tavi, P., and Ylä-Herttuala, S. (2012). AAV9-mediated VEGF-B Gene Transfer Improves Systolic Function in Progressive Left Ventricular Hypertrophy. Mol. Ther. *20*, 2212–2221. https://doi.org/10.1038/mt.2012.145.

16. Nurro, J., Halonen, P.J., Kuivanen, A., Tarkia, M., Saraste, A., Honkonen, K., Lähteenvuo, J., Rissanen, T.T., Knuuti, J., and Ylä-Herttuala, S. (2016). AdVEGF-B186 and AdVEGF-DΔNΔC induce angiogenesis and increase perfusion in porcine myocardium. Heart *102*, 1716–1720. https://doi.org/10.1136/heartjnl-2016-309373.

17. Korpela, H., Hätinen, O.-P., Nieminen, T., Mallick, R., Toivanen, P., Airaksinen, J., Valli, K., Hakulinen, M., Poutiainen, P., Nurro, J., and Ylä-Herttuala, S. (2021). Adenoviral VEGF-B186R127S gene transfer induces angiogenesis and improves perfusion in ischemic heart. iScience *24*, 103533. https://doi.org/10.1016/j.isci.2021.103533.

18. Mallick, R., Gurzeler, E., Toivanen, P.I., Nieminen, T., and Ylä-Herttuala, S. (2022). Novel Designed Proteolytically Resistant VEGF-B186R127S Promotes Angiogenesis in Mouse Heart by Recruiting Endothelial Progenitor Cells. Front. Bioeng. Biotechnol. *10*, 907538. https://doi.org/10.3389/fbioe.2022.907538.

19. Ylä-Herttuala, S., and Baker, A.H. (2017). Cardiovascular Gene Therapy: Past, Present, and Future. Mol. Ther. *25*, 1095–1106. https://doi.org/10.1016/j.ymthe.2017.03.027.

20. Kivelä, R., Bry, M., Robciuc, M.R., Räsänen, M., Taavitsainen, M., Silvola, J.M.U., Saraste, A., Hulmi, J.J., Anisimov, A., Mäyränpää, M.I., et al. (2014). VEGF-B-induced vascular growth leads to metabolic reprogramming and ischemia resistance in the heart. EMBO Mol. Med. *6*, 307–321. https://doi.org/10.1002/emmm.201303147.

21. Tirronen, A., Downes, N.L., Huusko, J., Laakkonen, J.P., Tuomainen, T., Tavi, P., Hedman, M., and Ylä-Herttuala, S. (2021). The Ablation of VEGFR-1 Signaling Promotes Pressure Overload-Induced Cardiac Dysfunction and Sudden Death. Biomolecules *11*, 452. https://doi.org/10.3390/biom11030452.

22. Plank, M.J., Sleeman, B.D., and Jones‡, P.F. (2004). The Role of the Angiopoietins in Tumour Angiogenesis. Growth Factors *22*, 1–11. https://doi.org/10.1080/08977190310001643218.

23. Akwii, R.G., Sajib, M.S., Zahra, F.T., and Mikelis, C.M. (2019). Role of Angiopoietin-2 in Vascular Physiology and Pathophysiology. Cells *8*, 471. https://doi.org/10.3390/cells8050471.

24. Battson, M.L., Lee, D.M., and Gentile, C.L. (2017). Endoplasmic reticulum stress and the development of endothelial dysfunction. Am. J. Physiol. Heart Circ. Physiol. *312*, H355–H367. https://doi.org/10.1152/ajpheart.00437.2016.

25. Chakraborty, R., Baek, J.H., Bae, E.Y., Kim, W.-Y., Lee, S.Y., and Kim, M.G. (2016). Comparison and contrast of plant, yeast, and mammalian ER stress and UPR. Appl. Biol. Chem. *59*, 337–347. https://doi.org/10.1007/s13765-016-0167-6.

26. Zhang, L., Zhang, C., and Wang, A. (2016). Divergence and Conservation of the Major UPR Branch IRE1-bZIP Signaling Pathway across Eukaryotes. Sci. Rep. *6*, 27362. https://doi.org/10.1038/srep27362.

27. Huusko, J., Merentie, M., Dijkstra, M.H., Ryhänen, M.M., Karvinen, H., Rissanen, T.T., Vanwildemeersch, M., Hedman, M., Lipponen, J., Heinonen, S.E., et al. (2010). The effects of VEGF-R1 and VEGF-R2 ligands on angiogenic responses and left ventricular function in mice. Cardiovasc. Res. *86*, 122–130. https://doi.org/10.1093/cvr/cvp382.

28. Mallick, R., and Ylä-Herttuala, S. (2022). Therapeutic Potential of VEGF-B in Coronary Heart Disease and Heart Failure: Dream or Vision? Cells *11*, 4134. https://doi.org/10.3390/cells11244134.

29. White, I.A., Sanina, C., Balkan, W., and Hare, J.M. (2016). Mesenchymal Stem Cells in Cardiology. Methods Mol. Biol. *1416*, 55–87. https://doi.org/10.1007/978-1-4939-3584-0_4.

30. Ren, J., Bi, Y., Sowers, J.R., Hetz, C., and Zhang, Y. (2021). Endoplasmic reticulum stress and unfolded protein response in cardiovascular diseases. Nat. Rev. Cardiol. *18*, 499–521. https://doi.org/10.1038/s41569-021-00511-w.

31. Song, M., and Cubillos-Ruiz, J.R. (2019). Endoplasmic Reticulum Stress Responses in Intratumoral Immune Cells: Implications for Cancer Immunotherapy. Trends Immunol. *40*, 128–141. https://doi.org/10.1016/j.it.2018.12.001.

32. Ata, R., and Antonescu, C.N. (2017). Integrins and Cell Metabolism: An Intimate Relationship Impacting Cancer. Int. J. Mol. Sci. *18*, 189. https://doi.org/10.3390/ijms18010189.

33. Banerjee, K., Keasey, M.P., Razskazovskiy, V., Visavadiya, N.P., Jia, C., and Hagg, T. (2017). Reduced FAK-STAT3 signaling contributes to ER stress-induced mitochondrial dysfunction and death in endothelial cells. Cell. Signal. *36*, 154–162. https://doi.org/10.1016/j.cellsig.2017.05.007.

34. Van Hove, I., Hu, T.-T., Beets, K., Van Bergen, T., Etienne, I., Stitt, A.W., Vermassen, E., and Feyen, J.H.M. (2021). Targeting RGD-binding integrins as an integrative therapy for diabetic retinopathy and neovascular age-related macular degeneration. Prog. Retin. Eye Res. *85*, 100966. https://doi.org/10.1016/j.preteyeres.2021.100966.

35. Chen, X., Shi, C., He, M., Xiong, S., and Xia, X. (2023). Endoplasmic reticulum stress: molecular mechanism and therapeutic targets. Signal Transduct. Target. Ther. *8*, 352. https://doi.org/10.1038/s41392-023-01570-w.

36. Mezu-Ndubuisi, O.J., and Maheshwari, A. (2021). The role of integrins in inflammation and angiogenesis. Pediatr. Res. *89*, 1619–1626. https://doi.org/10.1038/s41390-020-01177-9.

37. Javid, H., Oryani, M.A., Rezagholinejad, N., Esparham, A., Tajaldini, M., and Karimi-Shahri, M. (2024). RGD peptide in cancer targeting: Benefits, challenges, solutions, and possible integrin-RGD interactions. Cancer Med. *13*, e6800. https://doi.org/10.1002/cam4.6800.

38. Sultan, I., Ramste, M., Peletier, P., Hemanthakumar, K.A., Ramanujam, D., Tirronen, A., von Wright, Y., Antila, S., Saharinen, P., Eklund, L., et al. (2024). Contribution of VEGF-B-Induced Endocardial Endothelial Cell Lineage in Physiological Versus Pathological Cardiac Hypertrophy. Circ. Res. *134*, 1465–1482. https://doi.org/10.1161/CIRCRESAHA.123.324136.

39. Natori, T., Sata, M., Washida, M., Hirata, Y., Nagai, R., and Makuuchi, M. (2002). G-CSF stimulates angiogenesis and promotes tumor growth: Potential contribution of bone marrow-derived endothelial progenitor cells. Biochem. Biophys. Res. Commun. *297*, 1058–1061. https://doi.org/10.1016/S0006-291X(02)02335-5.

40. Cho, H.-J., Kim, H.-S., Lee, M.-M., Kim, D.-H., Yang, H.-J., Hur, J., Hwang, K.-K., Oh, S., Choi, Y.-J., Chae, I.-H., et al. (2003). Mobilized Endothelial Progenitor Cells by Granulocyte-Macrophage Colony-Stimulating Factor Accelerate Reendothelialization and Reduce Vascular Inflammation After Intravascular Radiation. Circulation *108*, 2918–2925. https://doi.org/10.1161/01.CIR.0000097001.79750.78.

41. Kovacic, J.C., Muller, D.W.M., and Graham, R.M. (2007). Actions and therapeutic potential of G-CSF and GM-CSF in cardiovascular disease. J. Mol. Cell. Cardiol. *42*, 19–33. https://doi.org/10.1016/j.yjmcc.2006.10.001.

42. Ying, R., Li, S.W., Chen, J.Y., Zhang, H.F., Yang, Y., Gu, Z.J., Chen, Y.X., and Wang, J.F. (2018). Endoplasmic reticulum stress in perivascular adipose tissue promotes destabilization of atherosclerotic plaque by regulating GM-CSF paracrine. J. Transl. Med. *16*, 105. https://doi.org/10.1186/s12967-018-1481-z.

43. Grote, K., Schütt, H., and Schieffer, B. (2011). Toll-Like Receptors in Angiogenesis. ScientificWorldJournal. *11*, 981–991. https://doi.org/10.1100/tsw.2011.92.

44. Pereira, E. (2012). Contribution of the Unfolded Protein Response to VEGF Expression. https://doi.org/10.21007/etd.cghs.2012.0243.

45. Ghosh, R., Lipson, K.L., Sargent, K.E., Mercurio, A.M., Hunt, J.S., Ron, D., and Urano, F. (2010). Transcriptional regulation of VEGF-A by the unfolded protein response pathway. PLoS One *5*, e9575. https://doi.org/10.1371/journal.pone.0009575.

46. Yoshida, H., Matsui, T., Yamamoto, A., Okada, T., and Mori, K. (2001). XBP1 mRNA is induced by ATF6 and spliced by IRE1 in response to ER stress to produce a highly active transcription factor. Cell *107*, 881–891. https://doi.org/10.1016/S0092-8674(01)00611-0.

47. Liang, H., Xiao, J., Zhou, Z., Wu, J., Ge, F., Li, Z., Zhang, H., Sun, J., Li, F., Liu, R., and Chen, C. (2018). Hypoxia induces MIR-153 through the IRE1α-XBP1 pathway to fine tune the HIF1α/VEGFA axis in breast cancer angiogenesis. Oncogene *37*, 1961–1975. https://doi.org/10.1038/s41388-017-0089-8.

48. Duan, Q., Ni, L., Wang, P., Chen, C., Yang, L., Ma, B., Gong, W., Cai, Z., Zou, M.H., and Wang, D.W. (2016). Deregulation of XBP1 expression contributes to myocardial vascular endothelial growth factor-A expression and angiogenesis during cardiac hypertrophy *in vivo*. Aging Cell *15*, 625–633. https://doi.org/10.1111/acel.12460.

49. Zhang, S.X., Ma, J.H., Bhatta, M., Fliesler, S.J., and Wang, J.J. (2015). The unfolded protein response in retinal vascular diseases: Implications and therapeutic potential beyond protein folding. Prog. Retin. Eye Res. *45*, 111–131. https://doi.org/10.1016/j.preteyeres.2014.12.001.

50. Cai, H., Gong, L., Liu, J., Zhou, Q., and Zheng, Z. (2019). Diosgenin inhibits tumor angiogenesis through regulating GRP78-mediated HIF-1α and VEGF/VEGFR signaling pathways. Pharmazie *74*, 680–684. https://doi.org/10.1691/ph.2019/9526.

51. Tissarinen, P., Tiensuu, H., Haapalainen, A.M., Määttä, T.A., Ojaniemi, M., Hallman, M., and Rämet, M. (2023). Elevated human placental heat shock protein 5 is associated with spontaneous preterm birth. Pediatr. Res. *94*, 520–529. https://doi.org/10.1038/s41390-023-02501-9.

52. Wang, D., Hou, C., Cao, Y., Cheng, Q., Zhang, L., Li, H., Feng, L., and Shen, Y. (2018). XBP1 activation enhances MANF expression via binding to endoplasmic reticulum stress response elements within MANF promoter region in hepatitis B. Int. J. Biochem. Cell Biol. *99*, 140–146. https://doi.org/10.1016/j.biocel.2018.04.007.

53. Lähteenvuo, J., Hätinen, O.-P., Kuivanen, A., Huusko, J., Paananen, J., Lähteenvuo, M., Nurro, J., Hedman, M., Hartikainen, J., Laham-Karam, N., et al. (2020). Susceptibility to Cardiac Arrhythmias and Sympathetic Nerve Growth in VEGF-B Overexpressing Myocardium. Mol. Ther. *28*, 1731–1740. https://doi.org/10.1016/j.ymthe.2020.03.011.

54. Lõhelaid, H., Anttila, J.E., Liew, H.-K., Tseng, K.-Y., Teppo, J., Stratoulias, V., and Airavaara, M. (2022). UPR Responsive Genes Manf and Xbp1 in Stroke. Front Cell Neurosci. *16*, 900725. https://doi.org/10.3389/fncel.2022.900725.

55. Okada, K.I., Minamino, T., Tsukamoto, Y., Liao, Y., Tsukamoto, O., Takashima, S., Hirata, A., Fujita, M., Nagamachi, Y., Nakatani, T., et al. (2004). Prolonged endoplasmic reticulum stress in hypertrophic and failing heart after aortic constriction: Possible contribution of endoplasmic reticulum stress to cardiac myocyte apoptosis. Circulation *110*, 705–712. https://doi.org/10.1161/01.CIR.0000137836.95625.D4.

56. Karpanen, T., Bry, M., Ollila, H.M., Seppänen-Laakso, T., Liimatta, E., Leskinen, H., Kivelä, R., Helkamaa, T., Merentie, M., Jeltsch, M., et al. (2008). Overexpression of Vascular Endothelial Growth Factor-B in Mouse Heart Alters Cardiac Lipid Metabolism and Induces Myocardial Hypertrophy. Circ. Res. *103*, 1018–1026. https://doi.org/10.1161/CIRCRESAHA.108.178459.

57. Tirronen, A., Downes, N.L., Huusko, J., Laakkonen, J.P., Tuomainen, T., Tavi, P., Hedman, M., and Ylä-Herttuala, S. (2021). The ablation of vegfr-1 signaling promotes pressure overload-induced cardiac dysfunction and sudden death. Biomolecules *11*, 452. https://doi.org/10.3390/biom11030452.

58. Rissanen, T.T., Nurro, J., Halonen, P.J., Tarkia, M., Saraste, A., Rannankari, M., Honkonen, K., Pietilä, M., Leppänen, O., Kuivanen, A., et al. (2013). The bottleneck stent model for chronic myocardial ischemia and heart failure in pigs. Am. J. Physiol. Heart Circ. Physiol. *305*, H1297–H1308. https://doi.org/10.1152/ajpheart.00561.2013.

59. Hedman, M., Hartikainen, J., Syvänne, M., Stjernvall, J., Hedman, A., Kivelä, A., Vanninen, E., Mussalo, H., Kauppila, E., Simula, S., et al. (2003). Safety and feasibility of catheter-based local intracoronary vascular endothelial growth factor gene transfer in the prevention of postangioplasty and in-stent restenosis and in the treatment of chronic myocardial ischemia: Phase II results of the Kuopio angiogenesis trial (KAT). Circulation *107*, 2677–2683. https://doi.org/10.1161/01.cir.0000070540.80780.92.

60. Mäkinen, K., Mannine, H., Hedman, M., Matsi, P., Mussalo, H., Alhava, E., and Ylä-Herttuala, S. (2002). Increased vascularity detected by digital subtraction angiography after VEGF gene transfer to human lower limb artery: A randomized, placebo-controlled, double-blinded phase II study. Mol. Ther. *6*, 127–133. https://doi.org/10.1006/mthe.2002.0638.

61. Pulkkinen, H.H., Kiema, M., Lappalainen, J.P., Toropainen, A., Beter, M., Tirronen, A., Holappa, L., Niskanen, H., Kaikkonen, M.U., Ylä-Herttuala, S., and Laakkonen, J.P. (2021). BMP6/TAZ-Hippo signaling modulates angiogenesis and endothelial cell response to VEGF. Angiogenesis *24*, 129–144. https://doi.org/10.1007/s10456-020-09748-4.

62. Kuroda, H., Tachikawa, M., Yagi, Y., Umetsu, M., Nurdin, A., Miyauchi, E., Watanabe, M., Uchida, Y., and Terasaki, T. (2019). Cluster of Differentiation 46 Is the Major Receptor in Human Blood-Brain Barrier Endothelial Cells for Uptake of Exosomes Derived from Brain-Metastatic Melanoma Cells (SK-Mel-28). Mol. Pharm. *16*, 292–304. https://doi.org/10.1021/acs.molpharmaceut.8b00985.

63. Dobin, A., Davis, C.A., Schlesinger, F., Drenkow, J., Zaleski, C., Jha, S., Batut, P., Chaisson, M., and Gingeras, T.R. (2013). STAR: Ultrafast universal RNA-seq aligner. Bioinformatics *29*, 15–21. https://doi.org/10.1093/bioinformatics/bts635.

64. Bolger, A.M., Lohse, M., and Usadel, B. (2014). Trimmomatic: A flexible trimmer for Illumina sequence data. Bioinformatics *30*, 2114–2120. https://doi.org/10.1093/bioinformatics/btu170.

65. Liao, Y., Smyth, G.K., and Shi, W. (2014). FeatureCounts: An efficient general purpose program for assigning sequence reads to genomic features. Bioinformatics *30*, 923–930. https://doi.org/10.1093/bioinformatics/btt656.

66. Zhu, A., Ibrahim, J.G., and Love, M.I. (2019). Heavy-Tailed prior distributions for sequence count data: Removing the noise and preserving large differences. Bioinformatics *35*, 2084–2092. https://doi.org/10.1093/bioinformatics/bty895.

67. Love, M.I., Huber, W., and Anders, S. (2014). Moderated estimation of fold change and dispersion for RNA-seq data with DESeq2. Genome Biol. *15*, 550. https://doi.org/10.1186/s13059-014-0550-8.

68. Blighe, K., Rana, S., and Lewis, M. (2018). EnhancedVolcano: Publication-ready volcano plots with enhanced colouring and labeling. https://github.com/kevinblighe/EnhancedVolcano.

69. Liberzon, A., Subramanian, A., Pinchback, R., Thorvaldsdóttir, H., Tamayo, P., and Mesirov, J.P. (2011). Molecular signatures database (MSigDB) 3.0. Bioinformatics *27*, 1739–1740. https://doi.org/10.1093/bioinformatics/btr260.

70. Subramanian, A., Tamayo, P., Mootha, V.K., Mukherjee, S., Ebert, B.L., Gillette, M.A., Paulovich, A., Pomeroy, S.L., Golub, T.R., Lander, E.S., and Mesirov, J.P. (2005). Gene set enrichment analysis: A knowledge-based approach for interpreting genome-wide expression profiles. Proc. Natl. Acad. Sci. USA *102*, 15545–15550. https://doi.org/10.1073/pnas.0506580102.

71. Wu, T., Hu, E., Xu, S., Chen, M., Guo, P., Dai, Z., Feng, T., Zhou, L., Tang, W., Zhan, L., et al. (2021). clusterProfiler 4.0: A universal enrichment tool for interpreting omics data. Innovation *2*, 100141. https://doi.org/10.1016/j.xinn.2021.100141.

72. Morgan, M., Falcon, S., and Gentleman, R. (2023). GSEABase: Gene set enrichment data structures and methods. R package version 1.64.0. https://bioconductor.org/packages/GSEABase.

73. Livak, K.J., and Schmittgen, T.D. (2001). Analysis of relative gene expression data using real-time quantitative PCR and the 2(-Delta Delta C(T)) Method. Methods *25*, 402–408.

74. Ramsay, E., Montaser, A.B., Niitsu, K., Urtti, A., Auriola, S., Huttunen, K.M., Uchida, Y., Kidron, H., and Terasaki, T. (2024). Transporter Protein Expression of Corneal Epithelium in Rabbit and Porcine: Evaluation of Models for Ocular Drug Transport Study. Mol. Pharm. *21*, 3204–3217. https://doi.org/10.1021/acs.molpharmaceut.3c01210.

75. Demichev, V., Messner, C.B., Vernardis, S.I., Lilley, K.S., and Ralser, M. (2020). DIA-NN: neural networks and interference correction enable deep proteome coverage in high throughput. Nat. Methods *17*, 41–44. https://doi.org/10.1038/s41592-019-0638-x.

YMTHE, Volume *33*

Supplemental Information

VEGF-B is a novel mediator of ER stress which induces cardiac angiogenesis via RGD-binding integrins independent of VEGFR1/NRP activities

Rahul Mallick, Ahmed B. Montaser, Henna Komi, Greta Juusola, Annakaisa Tirronen, Erika Gurzeler, Maria Barbiera, Petra Korpisalo, Tetsuya Terasaki, Tiina Nieminen, and Seppo Ylä-Herttuala

Supplemental

Supplemental materials and methods

Supplemental Tables

Table S1: *Animal groups in the study*

Group	Model types	Day 0	Day 6	Number of animals		
1	VEGFR1 TK$^{-/-}$ C57BL/6J male mice (12 weeks old)	Gene transfer, LVEF measurement,	LVEF, LVESV, LVEDV, plasma protein analysis and immunohistochemistry	Ad-CMV	Ad-VEGF-B186	Ad-VEGF-B186R127S
				**5**	**5**	**5**
2	wildtype C57BL/6J male mice (12 weeks old)	Gene transfer	Immunohistochemistry	Ad-CMV	Ad-VEGF-B186	Ad-VEGF-B186R127S
				**6**	**7**	**6**
3	wildtype female domestic pigs (3 months old)	Gene transfer	Immunohistochemistry	Ad-LacZ	Ad-VEGF-B186	Ad-VEGF-B186R127S
				**6**	**6**	**6**
4	Ischemic female domestic pigs (3 months old) [Ischemia induced fourteen days before the gene transfer by placing the bottleneck stent to the left anterior descending artery in angiographic guidance]	Gene transfer	Immunohistochemistry	Ad-LacZ	Ad-VEGF-B186	Ad-VEGF-B186R127S
				**5**	**5**	**5**

Table S2: *Primer pairs for quantitative real-time PCR*

Target genes	Sequence types	Strands
**Human *GAPDH***	**Forward**	GCAAGAGCACAAGAGGAAGA (Sense)
	**Reverse**	CTACATGGCAACTGTGAGGAG (AntiSense)
**Human *HSPA5***	**Forward**	GGTGGATCACAAGGTCAAGAG (Sense)
	**Reverse**	CTACCACGCCCAGCTAATTT (AntiSense)
**Human *ANGPT2***	**Forward**	ATCAGGACACACCACGAATG (Sense)
	**Reverse**	CATCCTCACGTCGCTGAATAA (AntiSense)
**Human *VEGFA***	**Forward**	GCTTACTCTCACCTGCTTCTG (Sense)
	**Reverse**	CTGTCATGGGCTGCTTCTT (AntiSense)
**Human *ATF6***	**Forward**	GGAGCCACTGAAGGAAGATAAG (Sense)
	**Reverse**	GTGCTGCTGGAAGCAATAAAG (AntiSense)
**Human *ERN1***	**Forward**	CCTCCGAGCCATGAGAAATAA (Sense)
	**Reverse**	GGGAAGCGAGATGTGAAGTAG (AntiSense)

**Human *EIF2AK3***	**Forward**	GGAAACGAGAGCCGGATTTAT (Sense)
	**Reverse**	TATGGCAGCTTCCTGTTCTTC (AntiSense)
**Human *CSF1***	**Forward**	GGAGACCTCGTGCCAAATTA (Sense)
	**Reverse**	CGCATGGTGTCCTCCATTAT (AntiSense)
**Human *CSF2***	**Forward**	GAGCTAGAAACTCAGGATGGTC (Sense)
	**Reverse**	TCTTCTGCCATGCCTGTATC (AntiSense)
**Human *CSF3***	**Forward**	TGTGTCCTTCCCTGCATTT (Sense)
	**Reverse**	TTACCTATCTACCTCCCAGTCC (AntiSense)
**Human *XBP1***	**Forward**	GAGACAGAGAGCCAAGCTAATG (Sense)
	**Reverse**	CAGGTTCTTCCTTCACTGAGAC (AntiSense)
**Human *FLT1***	**Forward**	TGACACTTTGATCCCTGATGG (Sense)
	**Reverse**	CACAGGTCAGAAGCCCTATTT (AntiSense)
**Human *ITGAV***	**Forward**	CGACAGGCTCACATTCTACTT (Sense)
	**Reverse**	GGACTCGAGACTCCTCTTATCT (AntiSense)
**Human *ITGA5***	**Forward**	GGTGGACCAGGAAGCTATTT (Sense)
	**Reverse**	GAACCAGGTTGATCAGGTACTC (AntiSense)

Table S3: *Differential gene expression analysis and gene set enrichment analysis results*

Table S4: *Summary of DIA-based protein identification and quantification*

Supplemental Figures

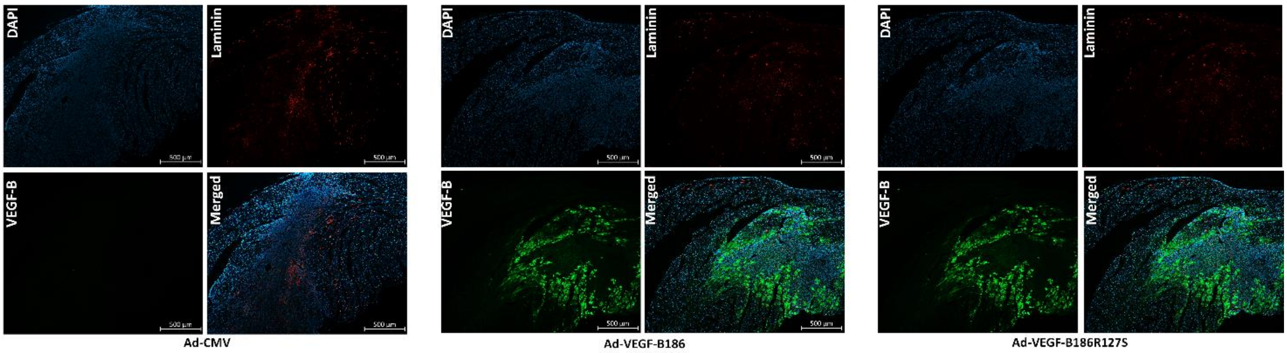

Figure S1: *VEGF-B expression in adenoviral vector transduced VEGFR1 TK $^{-/-}$ murine hearts.*

Representative images of heart tissue sections immunostained for VEGF-B, laminin, and DAPI following transduction with Ad-CMV, Ad-VEGF-B186, or Ad-VEGF-B186R127S. Scale bars: 500 µm.

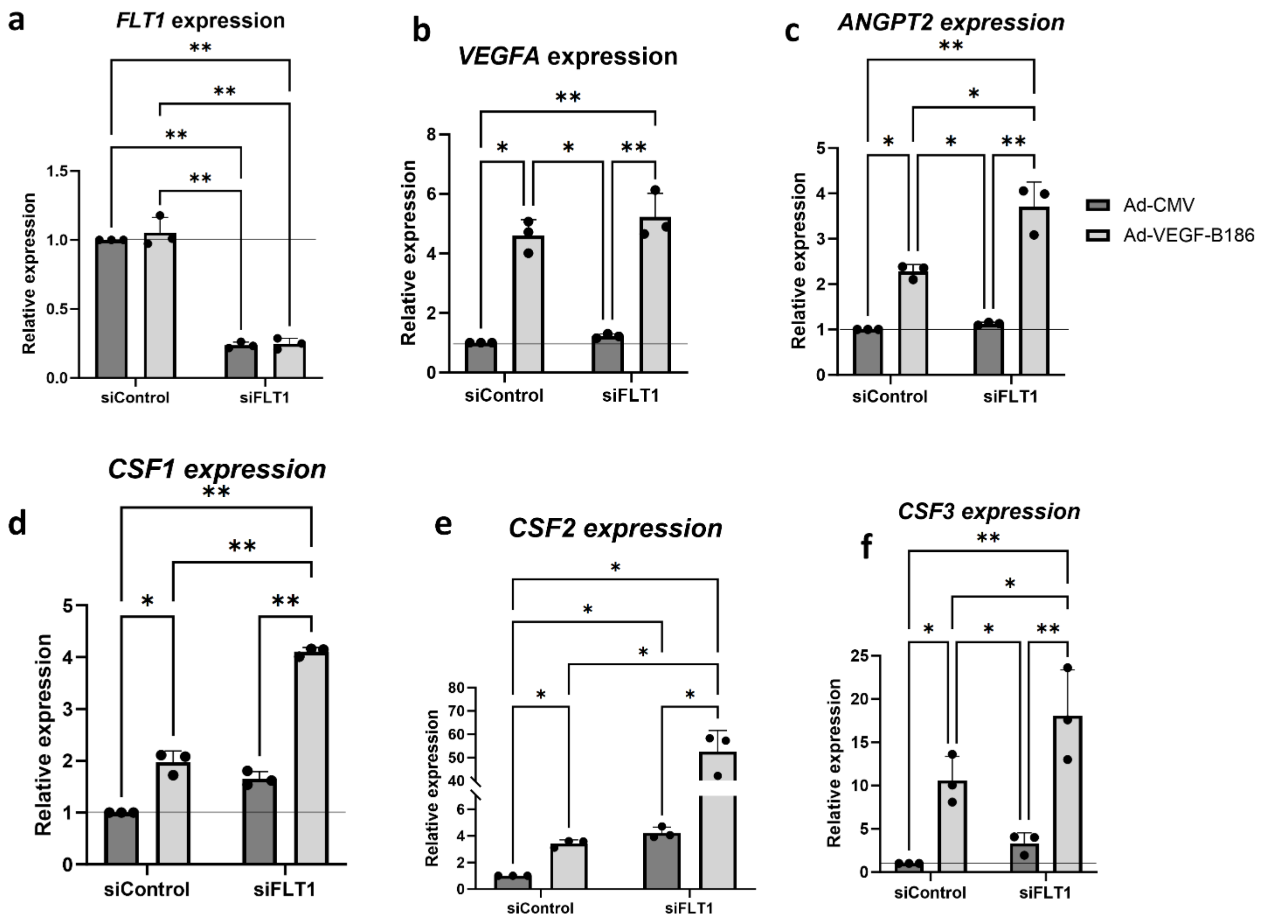

Figure S2: *VEGFR1 downstream signaling is not required for VEGF-B186-induced proangiogenic and haematopoietic growth inducing cytokine genes upregulation in cardiac microvascular endothelial cells*

a–f. Expression levels of *FLT1* (**a**), *VEGFA* (**b**), *ANGPT2* (**c**), *CSF1* (**d**), *CSF2* (**e**), and *CSF3* (**f**) in HMVEC-Cs treated with siControl (n=3) or siFLT1 (n=3) following Ad-VEGF-B186 or Ad-CMV transduction. The n value represents the number of individual cell culture experiments, which are defined as biological replicates. Horizontal bars indicate mean ± SD and *P* values versus each group by two-way ANOVA followed by Tukey's multiple comparison test. P value style: <0.05(*), <0.005(**).

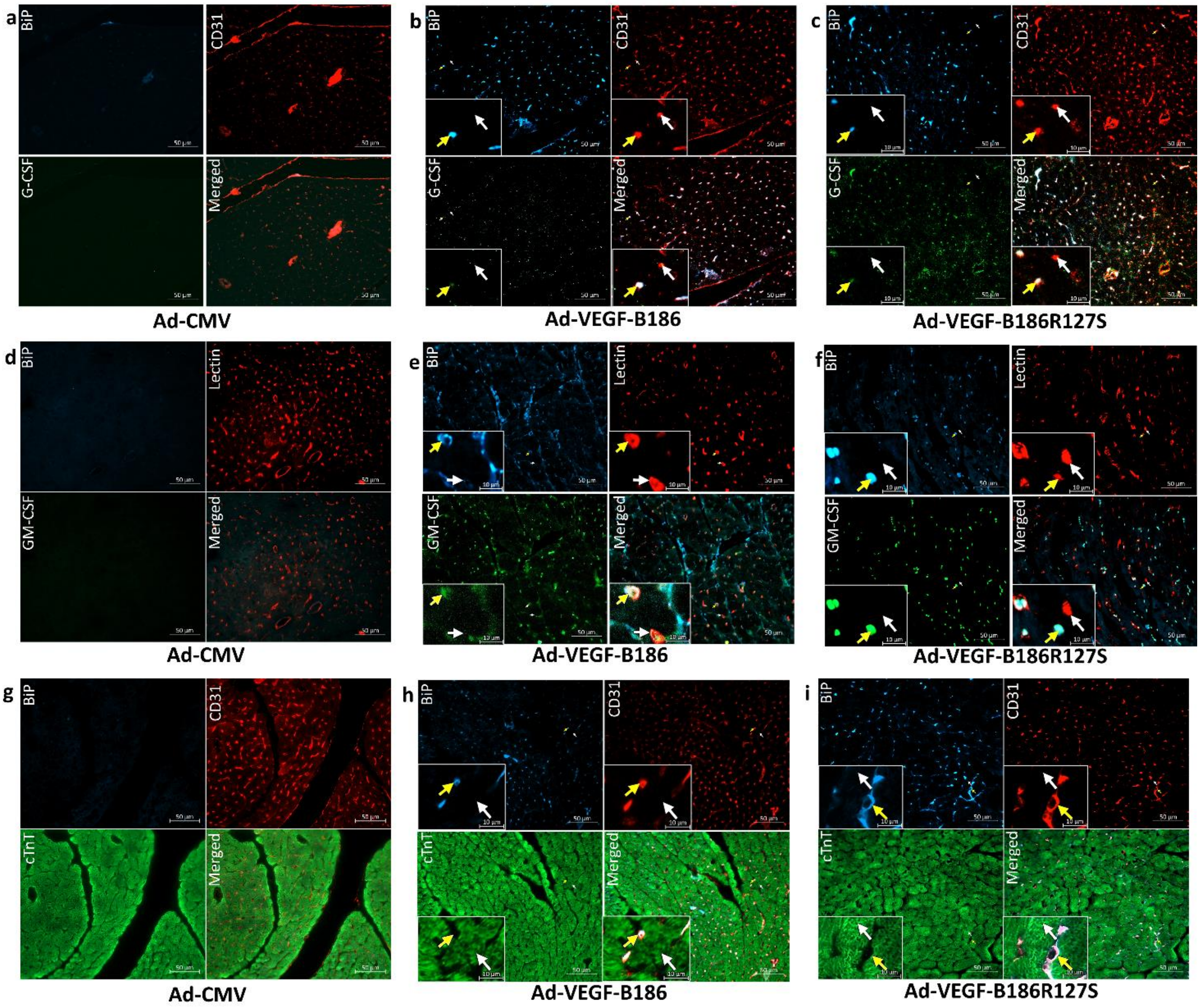

Figure S3: *Immunostained adenoviral vector transduced wildtype murine hearts.*

a–c. Representative images of sections stained for CD31, G-CSF, and BiP following Ad-CMV **(a)**, Ad-VEGF-B186 **(b)**, and Ad-VEGF-B186R127S **(c)** gene transfers. Yellow arrows indicate G-CSF–secreting, activated $BiP^{+}CD31^{+}$ endothelial cells, while white arrows indicate quiescent $CD31^{+}$ endothelial cells. **d–f.** Representative images of sections stained for lectin, GM-CSF, and BiP following Ad-CMV **(d)**, Ad-VEGF-B186 **(e)**, and Ad-VEGF-B186R127S **(f)** gene transfers. White arrows indicate $lectin^{+}$ endothelial cells, while yellow arrows indicate $lectin^{+}BiP^{+}GM\text{-}CSF^{+}$ activated endothelial cells. **g–i.** Representative images of sections stained for CD31, cTnT, and BiP following Ad-CMV **(g)**, Ad-VEGF-B186 **(h)**, and Ad-VEGF-B186R127S **(i)** gene transfers. White arrows denote $cTnT^{+}$ cardiomyocytes, while yellow arrows denote $BiP^{+}CD31^{+}$ endothelial cells. Scale bars: 50 µm (10 µm for the enlarged images).

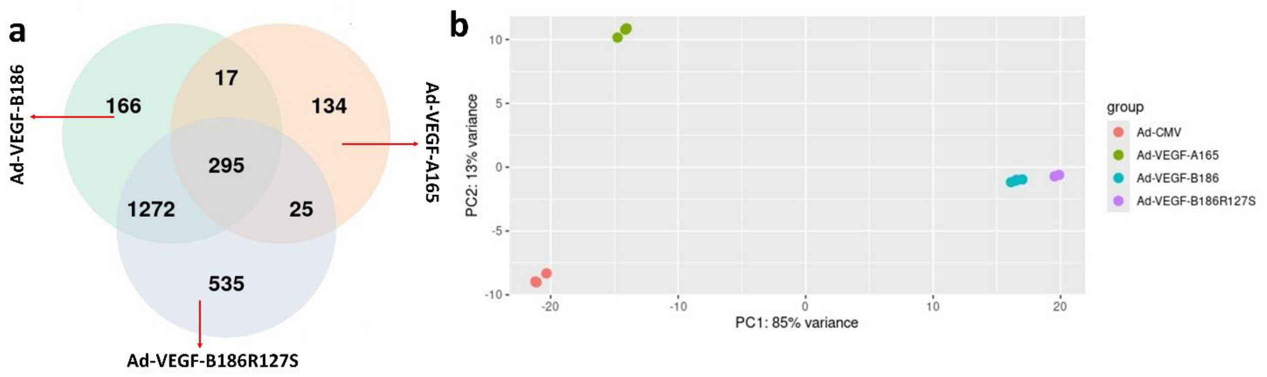

Figure S4: *Comparing differential gene expression in adenoviral vector transduced HMVEC-Cs*

Venn diagram (**a**) shows the overlap of differentially expressed genes across treatments. PCA plot (**b**) summarizes RNA-seq data distribution.

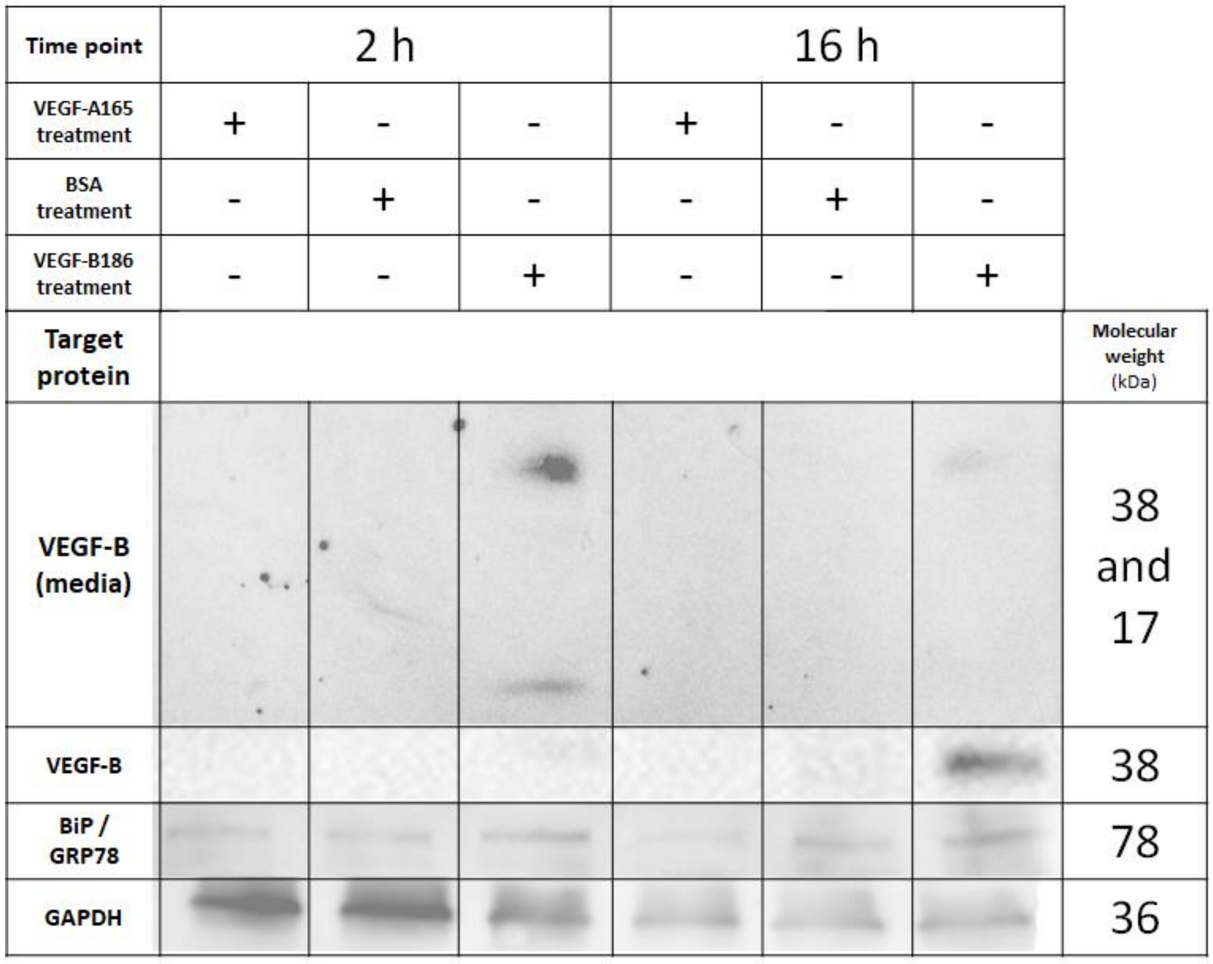

Figure S5: *rhVEGF-B186-induces expression of ER stress chaperon BiP/GRP78*

Immunoblot of VEGF-B (media and cell lysate), BiP / GRP78 and GAPDH expression in HUVECs 2 and 16 hrs post rhVEGF-B186, rhVEGF-A165 and BSA treatment

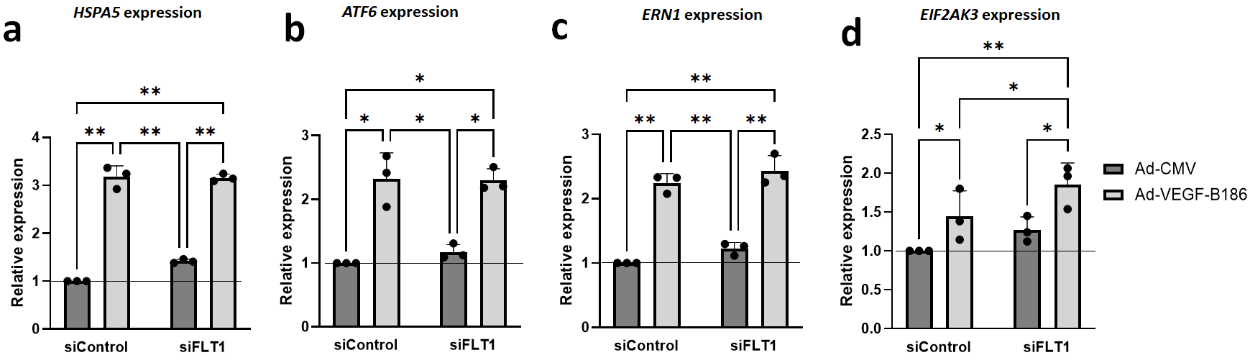

Figure S6: *VEGFR1 is dispensable for Ad-VEGF-B186-induced ER stress*

Expression of ER stress-related genes (**a-d**) is shown for siControl (n=3) or siFLT1 (n=3)-treated HMVEC-Cs post-transduction. The n value represents the number of individual cell culture experiments, which are defined as biological replicates. Horizontal bars indicate mean ± SD and *P* values versus each group by two-way ANOVA, followed by Tukey's multiple comparison test. P value style: <0.05(*), <0.005(**).

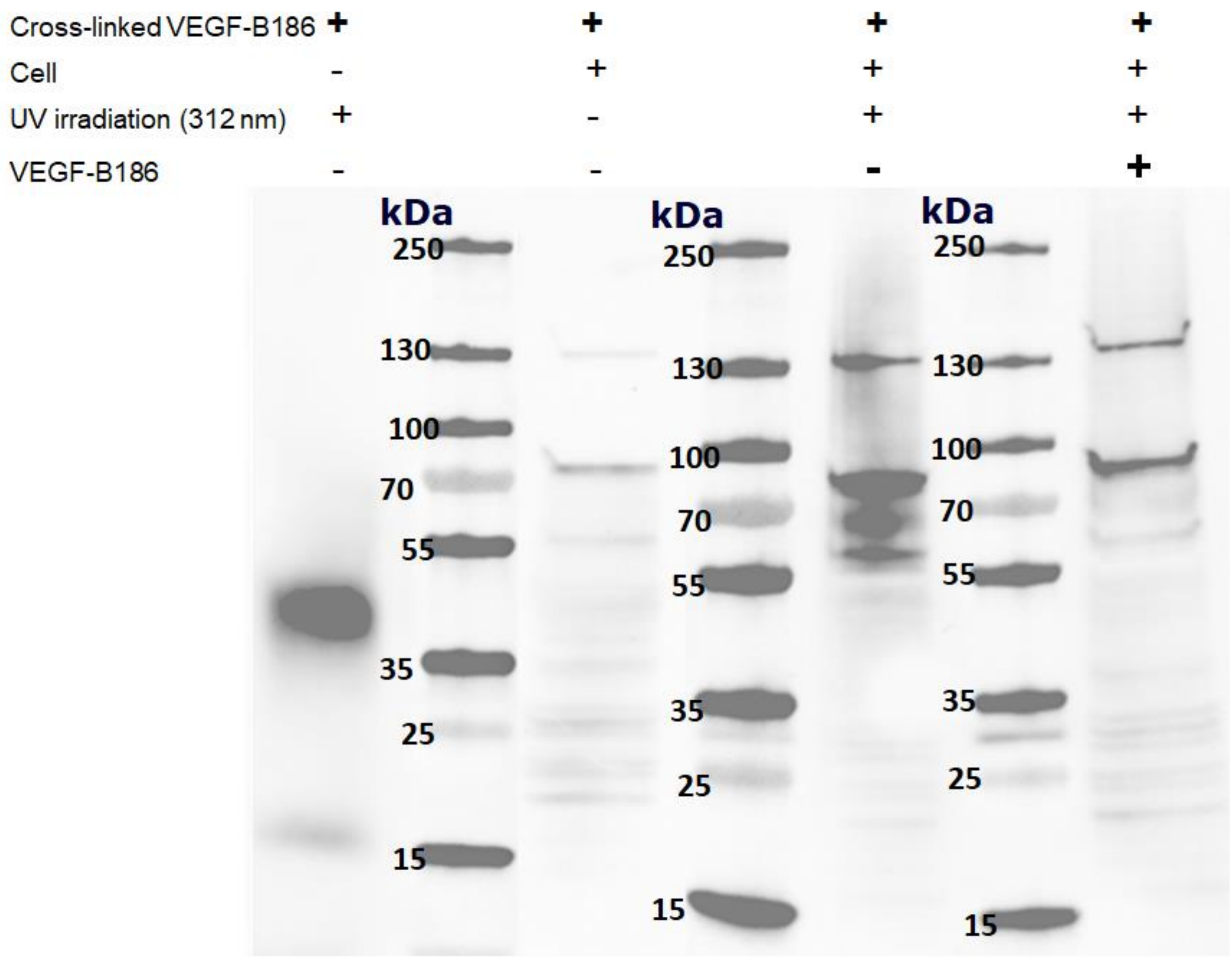

Figure S7: *The cross-linking method successfully identified the interactors of VEGF-B186.*

Biotin-labeled proteins that interact with VEGF-B186 in TeloHAECs are shown following SDS–PAGE separation and immunoblotting with HRP-conjugated streptavidin.

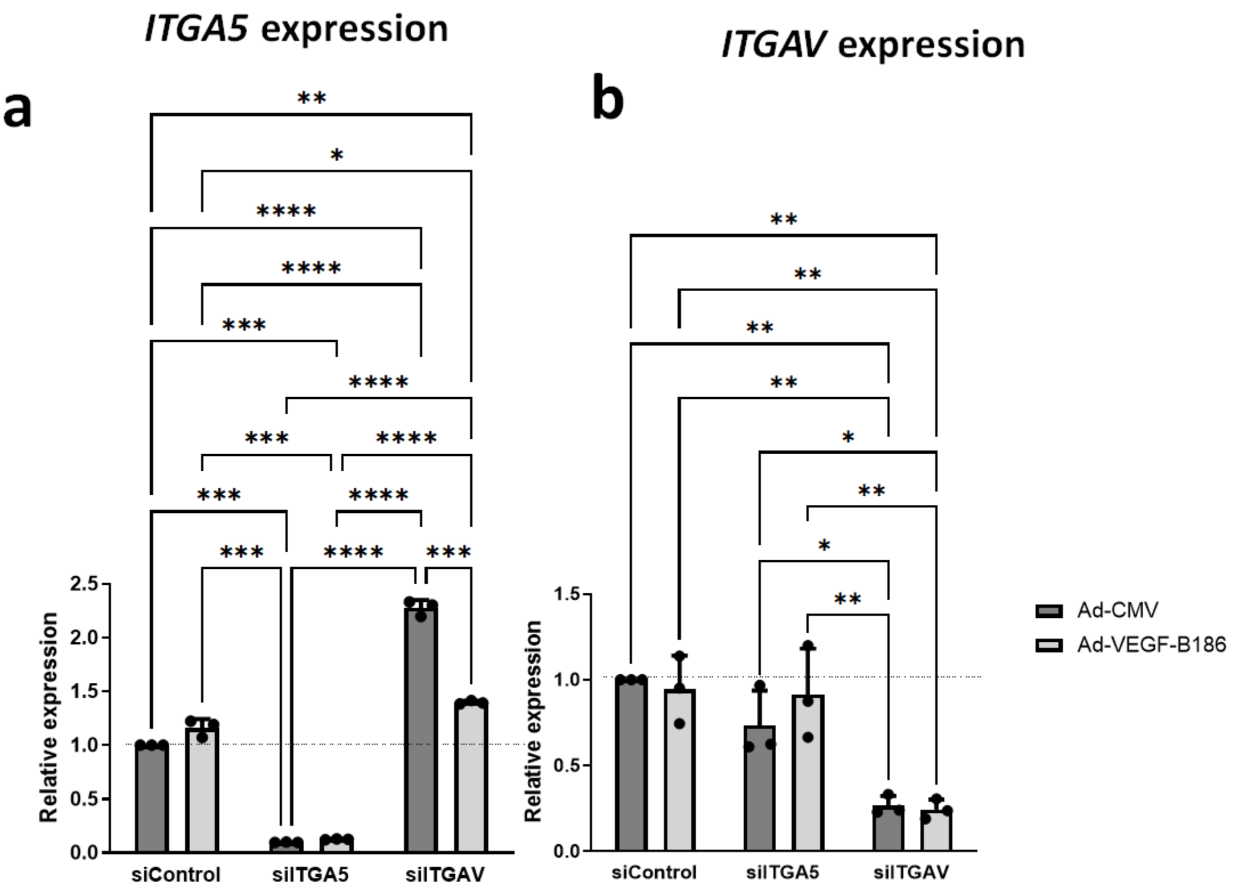

Figure S8: *ITGAV* and *ITGA5* silencing in endothelial cells.

RNA expression of *ITGA5* (**a**), and *ITGAV* (**b**) in siControl (n=3), siITGA5 (n=3) or siITGAV (n=3)-treated TeloHAECs after Ad-VEGF-B186 transduction. The n value represents the number of individual cell culture experiments, which are defined as biological replicates. Horizontal bars indicate mean ± SD and *P* values versus each group by two-way ANOVA followed by Tukey's multiple comparison test. P value style: <0.05(*), <0.005(**), <0.0005(***), <0.0001(****).

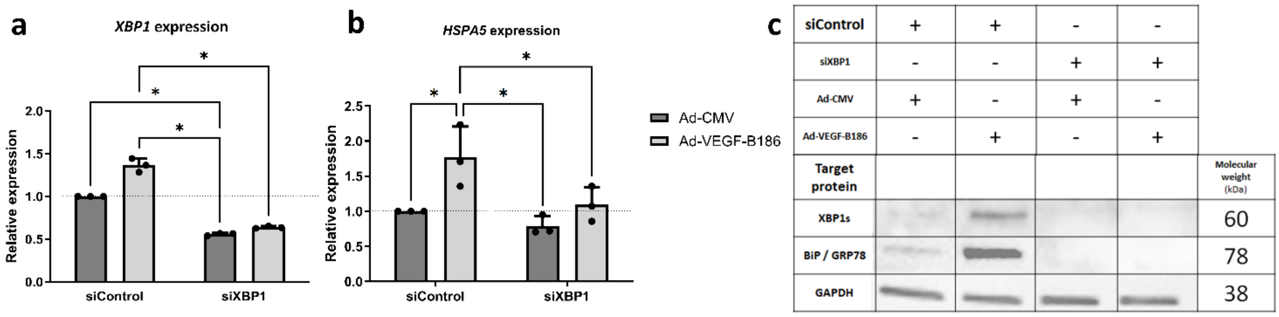

Figure S9: *The VEGF-B186-induced expression of ER stress chaperon HSPA5 is regulated by XBP1*

a,b. Graph comparing *XBP1* and *HSPA5* expression in HUVECs treated with either siControl or siXBP1 following transduction with Ad-VEGF-B186 or Ad-CMV. Horizontal bars indicate mean ± SD and *P* values versus each group by two-way ANOVA followed by Tukey's multiple comparison test. The n value represents the number of individual cell culture experiments, which are defined as biological replicates. P value style: <0.05(*), <0.005(**), <0.0005(***), <0.0001(****). **c.** Immunoblot showing the expression of XBP1s, BiP/GRP78, and GAPDH in HUVECs 24 hours after transduction with Ad-VEGF-B186, Ad-VEGF-A165, or Ad-CMV.
